# Whole Genome Characterisation of Non-O1/Non-O139 *Vibrio cholerae* in Wastewater Treatment Plants in the Tshwane District, South Africa

**DOI:** 10.3390/microorganisms14091995

**Published:** 2026-09-09

**Authors:** Onalenna Ramocha, Selinah Idah Mulaudzi, John Yenga Bolukaoto, Siesta Rashopole, Kholofelo Malemela, Setshaba Taukobong, Maphoshane Nchabeleng, Renee Street, Andrew Munyalo Musyoki

**Affiliations:** 1 Department of Microbiological Pathology, School of Medicine, Sefako Makgatho Health Sciences University, Pretoria 0204, South Africa; onalennaramocha@gmail.com (O.R.); selinah.mulaudzi@smu.ac.za (S.I.M.); john.bolukaoto@smu.ac.za (J.Y.B.); googlesiesta@yahoo.com (S.R.); kholofelo.malemela@smu.ac.za (K.M.); maphoshane.nchabeleng@smu.ac.za (M.N.); 2 National Health Laboratory Service, Dr George Mukhari Tertiary Laboratory, Pretoria 0208, South Africa; 3 Genomics Platform, South African Medical Research Council, Parrow, Cape Town 7505, South Africa; setshaba.taukobong@mrc.ac.za; 4 Environmental and Health Research Unit, South African Medical Research Council, Cape Town 7505, South Africa; renee.street@mrc.ac.za

**Keywords:** *V. cholerae*, non-O1/non-O139, whole genome sequencing, wastewater, South Africa

## Abstract

Non-O1/non-O139 *Vibrio cholerae* (NOVC) isolates are non-toxigenic and regarded as etiological agents of infrequent but mild-to-severe human gastroenteritis. In response to the 2022–2023 cholera outbreak in Tshwane, this study investigated the occurrence of NOVC isolates in wastewater within the Tshwane district, South Africa. A total of 341 wastewater samples were screened using Thiosulfate citrate bile salts sucrose (TCBS) media, with subsequent confirmation of *Vibrio cholerae* (*V. cholerae*) isolates by multiplex-PCR assays. Rep-PCR was performed to determine the genetic relatedness of the isolates. Selected isolates were subjected to whole genome sequencing (WGS). Of the 341 samples, PCR confirmed 143 isolates as NOVC. Genetic fingerprinting grouped these isolates into 12 distinct clusters, from which 12 representative isolates were selected for WGS and 10 isolates were confirmed as NOVC. The isolates harboured pathogenicity-related genes, such as *hlyA* and *rtxA*, as well as other genes contributing to bacterial adaptation such as *trkH* and *tnaA*. Drug resistance was mostly observed to first-line antibiotics, ciprofloxacin, chloramphenicol, and trimethoprim–sulfamethoxazole. Plasmid analysis showed six isolates harboured plasmids (*pA*, *p21L*, and *PVN84*) bearing multiple resistance determinants. Phylogenetic analysis showed evidence of genetic diversity amongst isolates. Although the isolates lacked classical toxigenic genes, they carried other virulence determinants associated with pathogenicity, posing a potential risk for clinical infection, highlighting the need for sustained surveillance.

## 1. Introduction

*Vibrio cholerae* (*V. cholerae*) is a curved, motile, Gram-negative organism found in marine habitats, which causes an acute, watery diarrheal disease known as cholera [1]. *Vibrio cholerae* may survive in a wide range of radically diverse conditions, including fresh and salty water; it can also live as cells that circulate freely or in biofilms on abiotic surfaces like zooplankton or phytoplankton, and it is capable of infecting the organism that hosts it [2]. Currently, about 200 different *V. cholerae* serogroups have been identified. However, O1 and O139 toxin-producing serogroups have been shown to be responsible for all significant known epidemics and pandemics [3,4]. Non-O1/non-O139 *V. cholerae* (NOVC) are isolates of *V. cholerae* that are unable to agglutinate with O1 or O139 antiserum. These isolates are typically non-toxigenic and regarded as etiological agents of infrequent but mild-to-severe human gastroenteritis [5]. Several investigations have documented the relevance of these strains in human infections [6,7], even though their clinical significance was previously disregarded [8]. Parenteral invasive infections and sporadic gastroenteritis are the most frequent infections of NOVC [9]. Due to the immediate results of genetic exchange processes, such as genetic recombination and horizontal gene transfer (HGT), NOVC strains are also thought to have the ability to aid in the formation of new virulent strains, including strains that can cause an epidemic [8]. In light of the recent cholera outbreak in Tshwane, it is therefore imperative to assess the potential public health risks posed by these NOVC isolates and to advise on surveillance programs in the district.

## 2. Materials and Methods

### 2.1. Study Area and Sample Collection

**Figure 1:**
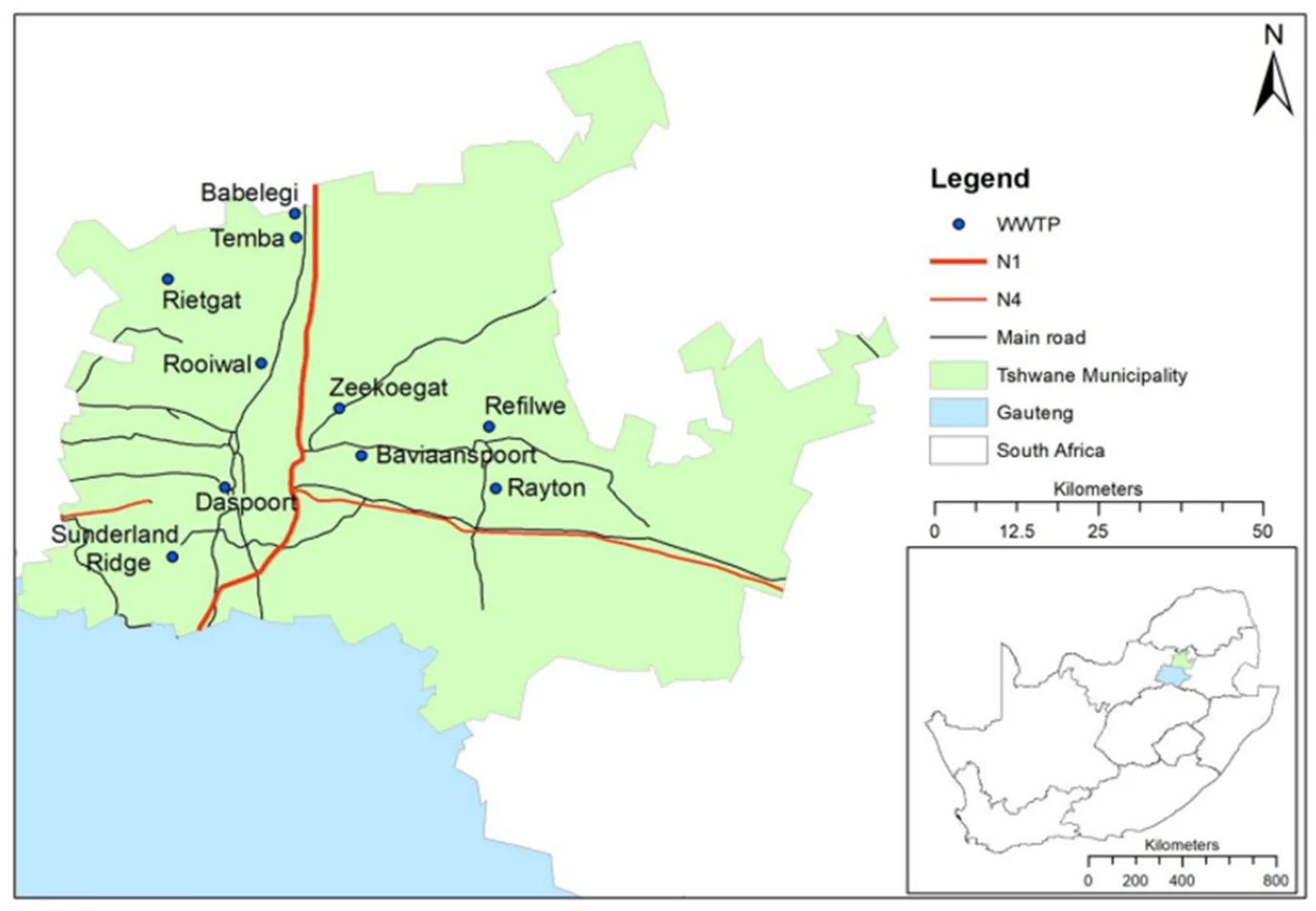
Geographic distribution of wastewater treatment plants in the Tshwane district where wastewater samples were collected. The map was produced using ArcGIS 10.6.1. Abbreviations: N1; National Route 1, N4; National Route 4.

Influent wastewater samples were collected from seven different wastewater treatment plants (WWTPs), namely, Babelegi, Temba, Rietgat, Daspoort, Sunderland Ridge, Zeekoegat, and Baviaanspoort, all within the Tshwane district, South Africa (Figure 1).

### 2.2. Identification of V. cholerae

The collected wastewater samples were enriched by inoculating 10 mL of each sample into 15 mL of alkaline peptone water (APW) (Thermo Fisher, Oxford, UK) and incubating at 35–37 °C for 24 h. Following incubation, the enriched samples were streaked onto TCBS (Thiosulfate citrate bile salts sucrose) agar (Thermo Fisher, UK) to facilitate the selective isolation of *Vibrio* species identified by yellow, smooth and shiny colonies [10]. The presumptive *V. cholerae* colonies were purified by sub-culturing on Mueller–Hinton agar (MHA) (Scharlab, Barcelona, Spain). The isolates were further characterised using Gram staining and oxidase test, with Gram-negative, comma-shaped, oxidase-positive colonies indicative of *V. cholerae* [10].

### 2.3. DNA Extraction and Genotypic Identification of V. cholerae

Genomic DNA of NOVC isolates was extracted using the boiling method [11]. Briefly, a bacterial suspension of a pure culture from a single colony was prepared in 1 mL of saline. The suspension was vortexed for 20 s and centrifuged at 13,000 rpm for 10 min. The supernatant was then resuspended in 200 µL of PCR-grade water. To extract DNA, the suspension was boiled on a heating block and ruptured using a cell disruptor. The suspension was centrifuged at 13,000 rpm for 10 min, after which the supernatant (200 µL) containing DNA was assessed using a Nanodrop™ LITE spectrophotometer (Thermo Scientific™, Waltham, MA, USA) for purity and concentration. Genotypic confirmation of the isolates was conducted using multiplex conventional PCR targeting the *ompW*, *ctxA*, and *tcpA* genes. *ompW* served as a conserved and species-specific marker commonly used for the identification of *V. cholerae*, whereas *ctxA* and *tcpA* genes were used to determine the presence of virulence genes associated with pathogenic *V. cholerae* strains [12], with the primer sequences shown in Supplementary Table S1. All primers used in this study were synthesised by Inqaba Biotechnical Industries (Pty) Ltd., Pretoria, South Africa. A 25 μL PCR reaction mix was prepared consisting of 12.5 μL MyTaq™ Red Mix (Bioline; London, UK), 0.5 μL of each primer (forward and reverse), 6.5 μL of PCR grade-water (BioConcept Ltd., Switzerland), and 5 μL of DNA template. Amplification was conducted in a T100™ Thermal cycler (Bio-Rad, Hercules, CA, USA), following the conditions shown in Supplementary Table S1. Amplicons were resolved by gel electrophoresis on a 2% agarose gel prepared in 1X Tris-Borate-EDTA buffer with 5 µL ethidium bromide (Promega, Madison, WI, USA) and run at 100 V for 60 min. DNA bands were visualised using the Gel Doc™ EZ system (Bio-Rad, Hercules, CA, USA).

### 2.4. Identification of Non-O1/Non-O139 V. cholerae

NOVC isolates were distinguished from O1/O139 strains using multiplex conventional PCR targeting rfb-O1- and rfb-O139-specific sequences. Isolates that showed no amplification for O1/O139 were subsequently subjected to further analysis. The target genes, primer sequences, amplicon sizes and optimised PCR conditions are listed in Supplementary Table S1. The PCR reaction mix was prepared as in Section 2.3 and amplified in a T100™ Thermal cycler (Bio-Rad, USA), using the conditions listed in Supplementary Table S1. Amplicons were detected and visualised as in Section 2.3.

### 2.5. Antimicrobial Susceptibility Testing

The Kirby–Bauer disc diffusion method was used to determine the antimicrobial susceptibility of non-O1/non-O139 *V. cholerae* isolates using Clinical Laboratory Standard Institute CLSI, 2024 guidelines. Antimicrobial susceptibility testing (AST) was performed using four antibiotic-impregnated discs containing meropenem, trimethoprim–sulfamethoxazole, ampicillin and levofloxacin (Mast Diagnostics, Merseyside, UK).

### 2.6. Repetitive Extragenic Palindromic PCR

Rep-PCR was performed to assess the genetic relatedness of the isolates. The primers and cycling conditions used for rep-PCR are listed in Supplementary Table S2. Amplification was conducted using the T100™ Thermal cycler (Bio-Rad, USA). Preparation of the reaction, amplicon detection and visualisation was conducted as in Section 2.3. Representative isolates from each cluster were subsequently subjected to whole genome sequencing (WGS) for further characterisation.

### 2.7. Whole Genome Sequencing

DNA was extracted using the Zymo Quick-DNA Fungal/Bacteria Miniprep kit (Zymo Research, Irvine, CA, USA). The resultant ultra-pure DNA was quality-checked and quantified using a Qubit 4.0 fluorometer (Thermo Fisher Scientific, Waltham, MA, USA). The Rapid Barcoding Kit 96 V14 (SQK-RBK114.96, Oxford, England) was used for library preparation following the NO-MISS protocol and loaded onto the Oxford Nanopore Technologies Minion R10.4.1 flow cell (FLO-MIN114, Oxford, England). Whole genome sequencing was performed on a Minion Mk1C sequencer platform (Oxford Nanopore Technology, England) for a 72 h run length and 200 bp minimum read length. Base-calling and demultiplexing of the raw FAST5 data were performed using Guppy v 6.5.7 with the super accuracy model through MinKNOW v24.02 software, which converted data to FASTQ format. The FASTQ files were subsequently uploaded to the cloud-based EPI2ME platform for further bioinformatics analysis.

### 2.8. Bioinformatics Analysis

FASTQ files were uploaded to the EPI2ME cloud-based platform and analysed using the wf-bacterial-genome workflow (https://epi2me.nanoporetech.com/epi2me-docs/workflows/wf-bacterial-genomes/ (accessed on 21 August 2026)). The quality control, filtering and trimming of the raw sequencing data was performed using Fastcat v0.18.6 (https://github.com/epi2me-labs/fastcat.git (accessed on 21 August 2026)) customised using the minimum_read_length of 1000 bp and a Fastlint quality threshold of 20 parameters. Sequencing reads with a quality score ≥8 were retained and concatenated for downstream analysis. Following quality filtering, de novo assembly of bacterial genomes was performed using Flye v2.9.5-b1801 [13], with the --nano-hq parameter. The assembled contigs were subsequently error-corrected and polished using Medaka v2.0.0 (https://github.com/nanoporetech/medaka.git (accessed on 21 August 2026)). Multilocus sequence typing (MLST) was done using MLST v2.23 (https://github.com/tseemann/mlst (accessed on 21 August 2026)). Prokka v1.14.5 (https://github.com/Prokka (accessed on 21 August 2026)) was used to annotate the resulting contigs. Antimicrobial resistance genes were identified using Resfinder v3.10 [14]. Genome mapping was constructed using prokseeversion 2.0.0 (https://proksee.ca/projects/new (accessed on 21 August 2026)). Resistance genes on a genome map constructed from proksee were predicted using the comprehensive antibiotic resistance database (CARD) resistance gene identifier (RGI) v6.0.3 [15]. Different virulence genes were predicted using proksee. Mobile genetic elements were predicted on proksee using mobileOG-db v1.1.3 [16]. Plasmid analysis was done on EPI2ME and, for profiling, the contigs belonging to different isolates were subjected to blast searches on the National Centre for Biotechnology Information (NCBI) database (https://blast.ncbi.nlm.nih.gov/Blast.cgi (accessed on 21 August 2026)) to identify plasmid sequences exhibiting the highest sequence similarity.

### 2.9. Phylogenetic Analysis

To investigate the evolutionary relationships among NOVC isolates, *gyrB* gene sequences from twelve locally obtained wastewater isolates were compared with corresponding sequences from ten reference isolates originating from different countries and retrieved from NCBI. Phylogenetic analysis was performed to determine the genetic relatedness of the local isolates to globally distributed NOVC strains. Sequence alignment using ClustalW and phylogenetic analysis was performed with MEGA version 12.0 (https://www.megasoftware.net (accessed on 21 August 2026)). Phylogenetic analysis was performed using the neighbour-joining method [17] based on the *gyrB* gene, a conserved housekeeping gene of *V. cholerae* with branch reliability assessed by 1000 bootstrap replicates [18]. Evolutionary distances were calculated using the Maximum Composite Likelihood method [19]. Positions with gaps or missing data were excluded by complete deletion, resulting in a final data set of 70 positions. All evolutionary analyses were conducted in MEGA12, utilising up to four parallel computing threads [20].

## 3. Results

### 3.1. Phenotypic and Molecular Identification of NOVC Isolates

A total of 341 wastewater samples were collected and analysed, and 319 yielded presumptive *V. cholerae* isolates. Among these, 192 were confirmed as *V. cholerae* by PCR targeting the *ompW* gene, with the *ctxA* and *tcpA* virulence genes detected in 52 and 51 isolates, respectively. Multiplex-PCR targeting the O1 and O139 serogroups identified 49 isolates positive for O1, while 143 isolates showed no amplification for either serogroup.

### 3.2. Antimicrobial Susceptibility Testing Results

**Figure 2:**
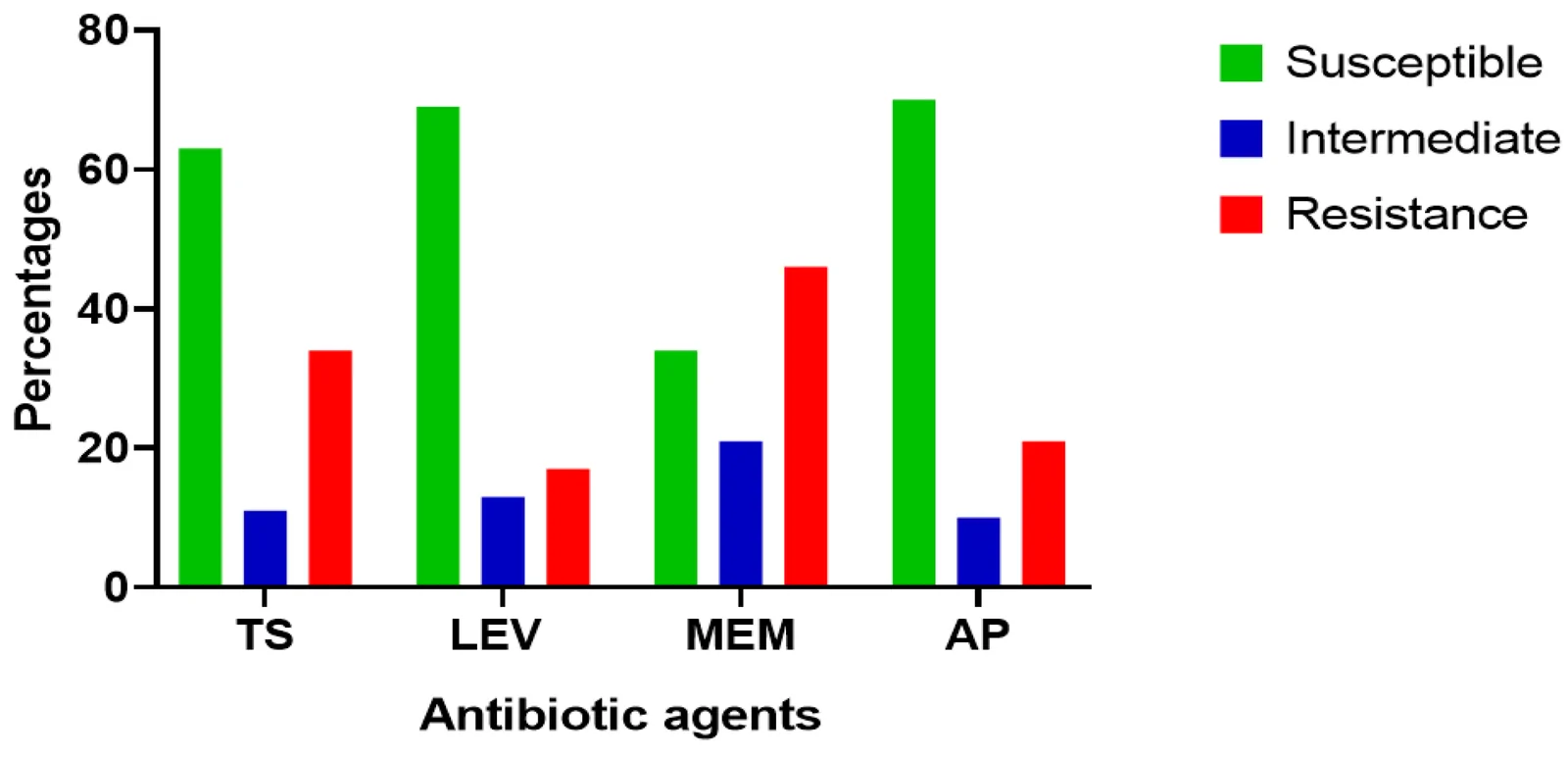
Antibiotic susceptibility trends of non-O1/non-O139 *V. cholerae* isolates recovered from wastewater samples, illustrating resistance and susceptibility patterns across different antibiotic agents (trimethoprim–sulfamethoxazole (TS), levofloxacin (LEV), meropenem (MEM), ampicillin (AP)).

A total of 63 non-O1/non-O139 *V. cholerae* isolates were tested against four antibiotics and are represented as percentages in Figure 2. Overall, most of the isolates displayed susceptibility to commonly used antibiotics, including trimethoprim–sulfamethoxazole (63%), levofloxacin (69%), and ampicillin (70%). However, a notable proportion of the isolates exhibited resistance to meropenem (46%), a last-resort carbapenem.

### 3.3. Repetitive Extragenic Palindromic PCR

**Figure 3:**
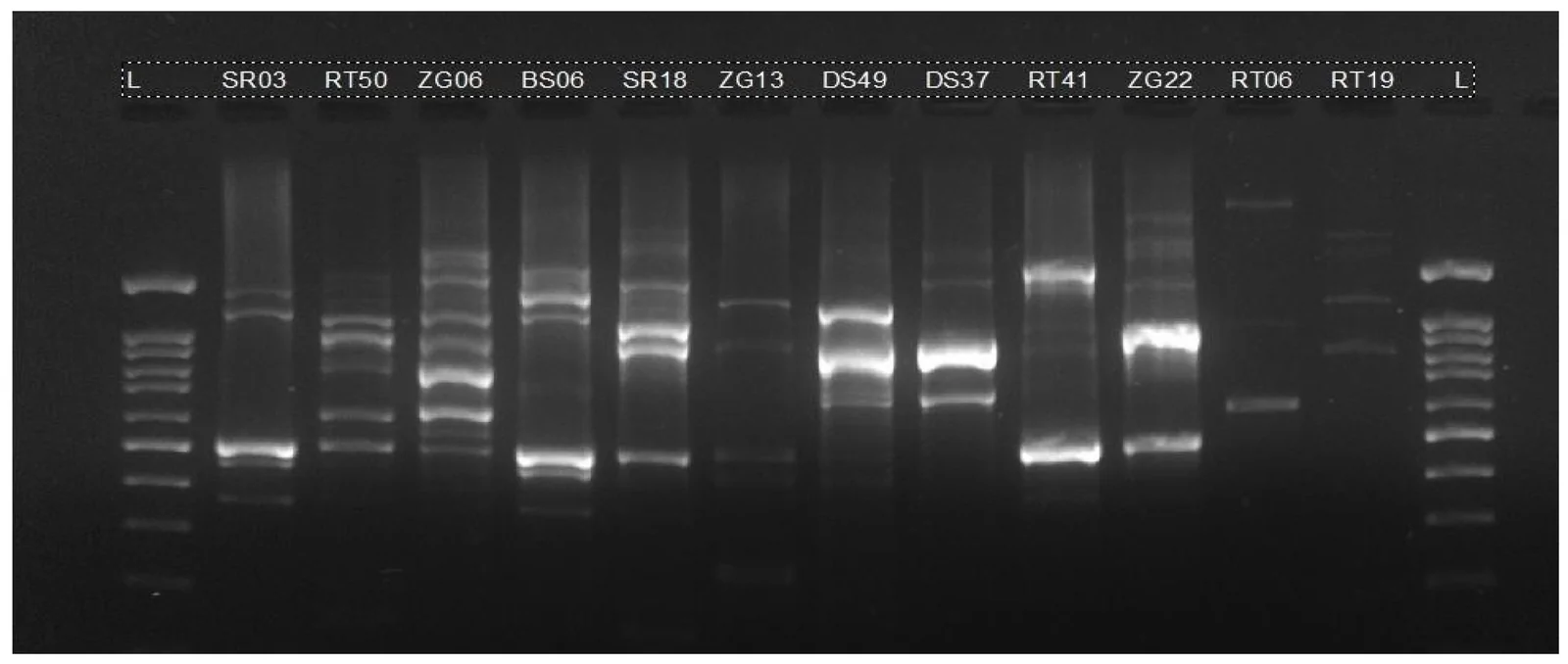
Agarose gel electrophoresis representing the genetic relatedness of non-O1/non-O139 *V. cholerae* isolates based on Rep-PCR analysis. The clustering illustrates the diversity and potential heterogeneity of isolates recovered from wastewater samples, highlighting distinct genetic groups among the strains.

The analysis clustered the strains into twelve distinct groups based on their genetic relatedness comprising of isolates from cluster I (SR03, DS48, and DS06), cluster II (RT50, SR46, ZG50, BS10, and SR41), cluster III (ZG06, BS37, SR37, and RT26), cluster IV (BS06, BS35, BB27, and DS19), cluster V (SR18 and ZG35), cluster VI (ZG13, BS25, and RT26), cluster VII (DS49, SR49, and BS49), cluster VIII (DS37, TB37, TB33, BS32, DS27, BS27, and RT27), cluster IX (RT41, TB46, and SR37), cluster X (ZG22, DS10, ZG42, and DS41), cluster XI (RT06, ZG27, and BS05) and cluster XII (RT19, RT46, ZG45, and RT10). As illustrated in Figure 3, the isolates demonstrated considerable genetic diversity, underscoring the heterogeneity of non-O1/non-O139 *V. cholerae* populations in wastewater environments, from which twelve representative isolates were selected for WGS and subsequently confirmed as NOVC.

### 3.4. Sequencing Quality Metrics

Several key quality metrics were observed and demonstrated the read count across the ten isolates ranging from 6160 to 85,840 reads, with the median read length ranging from 2401 to 4058.5 bp, while the number of contigs per assembly ranged from 3 to 86. The assemblies had an average N50 value of 4.68 kb and the minimum quality score of 8. The sequencing depth/coverage of the isolates is illustrated in Supplementary S2.

### 3.5. Whole Genome Characteristics of NOVC Isolates

The genomic characterisation of ten NOVC isolates generated using EPI2ME are presented in Table 1. Ten isolates were identified as *V. cholerae*, with the remaining two classified as Vibrio species. Genome sizes ranged from 3.73 to 4.92 Mb. No sequence types matched entries in the MLST database.

### 3.6. Genomic Characteristics of NOVC Isolates

Table 2 summarises the key genomic features of NOVC isolates, as predicted by the Proksee genome analysis softwareversion 2.0.0. The analysed genomes exhibit a total number of genes ranging from 3562 to 8446, with the CDS ranging from 3430 to 8302 and the GC (guanine + cytosine) content showing significant variation across the genomes. The number of sORFs (small open reading frames) ranged from 4672 to 9591, widely distributed throughout the chromosomes. For RNA elements, the number of tRNA (transfer RNA) and rRNA (ribosomal RNA) genes ranged from 88 to 109 and 20 to 34, respectively. tmRNA (transfer-messenger RNA) genes were present in nine out of ten isolates, and CRISPR elements were found in eight out of ten isolates. This overview provides insights into the genomic structure of the isolates.

### 3.7. Mobile Genetic Elements

Table 3 summarises functional categories consisting of various mobile genetic elements (MGEs) identified in NOVC isolates, which were analysed using MobileOG in Proksee software. Multiple functional categories were detected across all isolates, with the phage-related elements and DNA repair–recombination–repair elements being the most prevalent. These findings highlight the potential role of MGEs in genomic plasticity, virulence and the dissemination of antimicrobial resistance among environmental *V. cholerae* populations.

### 3.8. Plasmids

**Figure 4:**
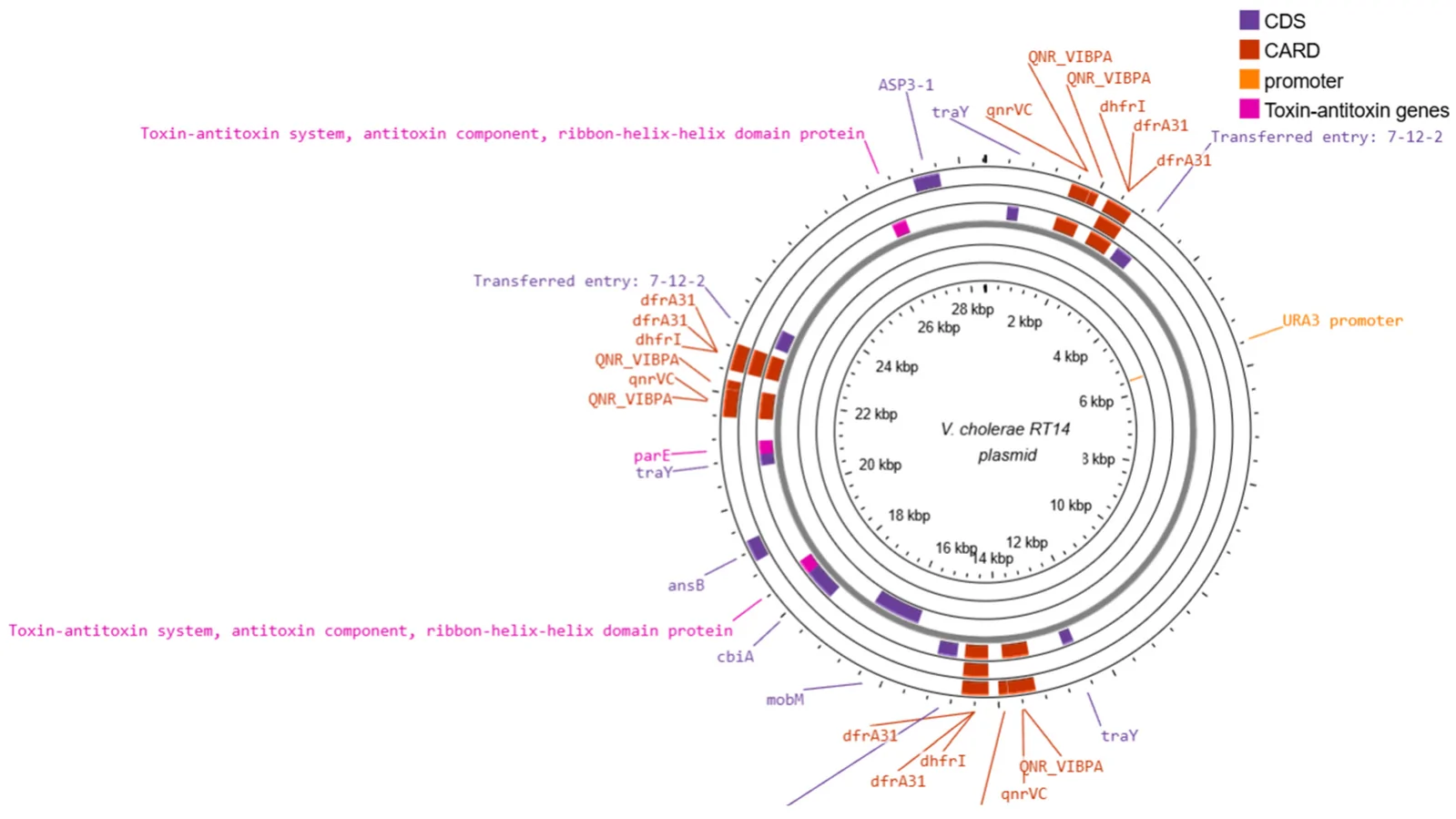
Circular map of a plasmid from the NOVC isolate RT14 highlighting CDSs, antimicrobial resistance determinants and toxin–antitoxin genes. Plasmid profiling was conducted based on BLAST analysis from the NCBI database and annotated using pLannotate and bakta on Proksee.

Plasmid analysis based on EPI2ME revealed that out of ten examined isolates, five harboured plasmids. The sizes of these plasmids ranged from 3903 bp to 16,000 bp. Among these, three distinct plasmid profiles (pA, PVN84 and p21L) were identified based on BLAST analysis from the NCBI database, including some unnamed plasmids. Most of the isolates in this study harboured plasmids which carried multiple resistance determinants, genes encoding toxin–antitoxin systems, and replicase and relaxases. Figure 4 and Supplementary S2 present circular plasmid maps indicating the distribution of CDS, CARD and toxin–antitoxin genes. These genes confer resistance mainly to trimethoprim and quinolones. The presence of these plasmids underscores the potential role of mobile genetic elements in disseminating antimicrobial resistance among environmental *V. cholerae* populations.

### 3.9. Antibiotic Resistance Profiles of NOVC Isolates

**Figure 5:**
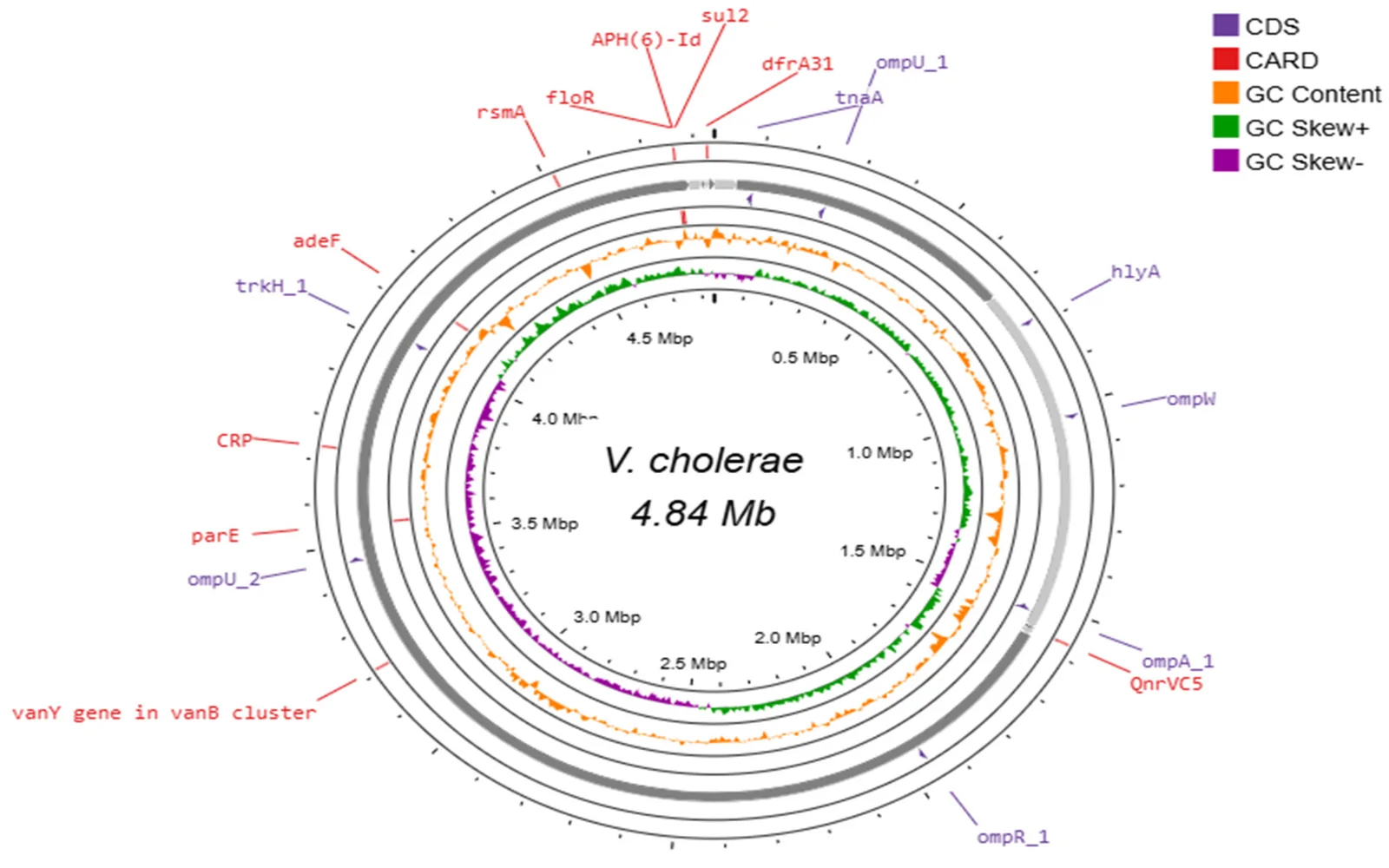
Complete genome annotation of the NOVC isolate BS12. The innermost rings represent the G + C content and GC skew, while the outermost rings display coding DNA sequences (CDSs) and antimicrobial resistant genes identified using CARD. CDSs homologous to known virulence genes (hlyA, gyrA/B, outer membrane proteins (ompW, ompV, ompA, ompR and ompH), trkH and tnaA) are highlighted, alongside CARD-annotated resistance genes (r/smA and CRP). This genomic map provides a visual overview of both the virulence potential and antimicrobial resistance profile of isolate BS12.

The analysis predicted 30 antibiotic resistance genes across the isolates, mostly associated with resistance mechanisms such as efflux pump, antibiotic inactivation, target protection, enzymatic degradation, enzymatic inactivation, antibiotic efflux, and target replacement. The predicted resistance genes conferred resistance against multiple drug classes such as aminoglycosides (streptomycin), cephalosporins (ceftazidime), fluoroquinolones (ciprofloxacin), tetracyclines (tetracycline), phenicols (chloramphenicol), folate pathway inhibitors (trimethoprim and sulfamethoxazole), macrolide (erythromycin) and beta-lactams (ampicillin, amoxicillin, and piperacillin) as detailed in Table 4. These genes were associated with resistance to various drug classes, including aminoglycosides (Alph (6)-id, Alph (3″)-Ib, rsmA), tetracyclines (TXR), beta-lactams (CARB-9), glycopeptides (VanT in the VanG cluster and VanY in the VanB cluster), fluoroquinolones (parE, qnrVC4, adeF), phenicols (flor and adeF), trimethoprim (dfrA31) and sulfonamides (sul2). The distribution and functional classification of these genes is illustrated in Figure 5 and Supplementary S2. These results highlight the diverse AMR potential present in environmental *V. cholerae* isolates.

### 3.10. Virulence-Associated Genes of NOVC Isolates

The virulence-associated genes identified in NOVC isolates were categorised by functional group. Cytolytic factors included hlyA, rtxA, and stn; adhesion and colonisation factors comprised mshA, ompU, tcpA, HA/P, and nagH; secretion system components included T3SS, T6SS, rtxC, VPI-1, and VPI-2; biofilm-associated genes comprised VSP1/VSP2, als, and makA; and regulatory/metabolic elements included toxR, VSPR, rfbv, rfbc, trkH, tnaA, gyrA, and gyrB. These results highlight the virulence potential present in environmental NOVC isolates. The distribution of the virulence genes in NOVC and the functional groups is depicted Table 5.

### 3.11. Circular Representation of a Whole Genome Map of NOVC Isolates

Genome maps of the ten NOVC isolates (B12, DS13, BS13, DS14, BS14, ZG14, RT14, SR14, DS12, ZG12, RT12, and SR12) were generated using Proksee (Supplementary S2). A circular map of one of the isolates is shown as a representation of the results obtained in Figure 5. The maps were annotated to display key genomic features, including virulence genes (CDS), resistance genes (CARD), G + C content, and GC skew. Notable virulence genes detected among the isolates included hlyA, gyrA/B, trkH, and tnaA, while resistance genes comprised rsmA, CRP, APH (6)-Id, floR, dfrA31, almF, and qnrVC4. The G + C content of these genomes ranged from 44% to 50%, reflecting variability among the isolates. These genomic analyses highlight both the virulence potential and antimicrobial resistance profiles of NOVC from wastewater.

### 3.12. Phylogenetic Analysis of the gyrB Gene in NOVC Isolates

**Figure 6:**
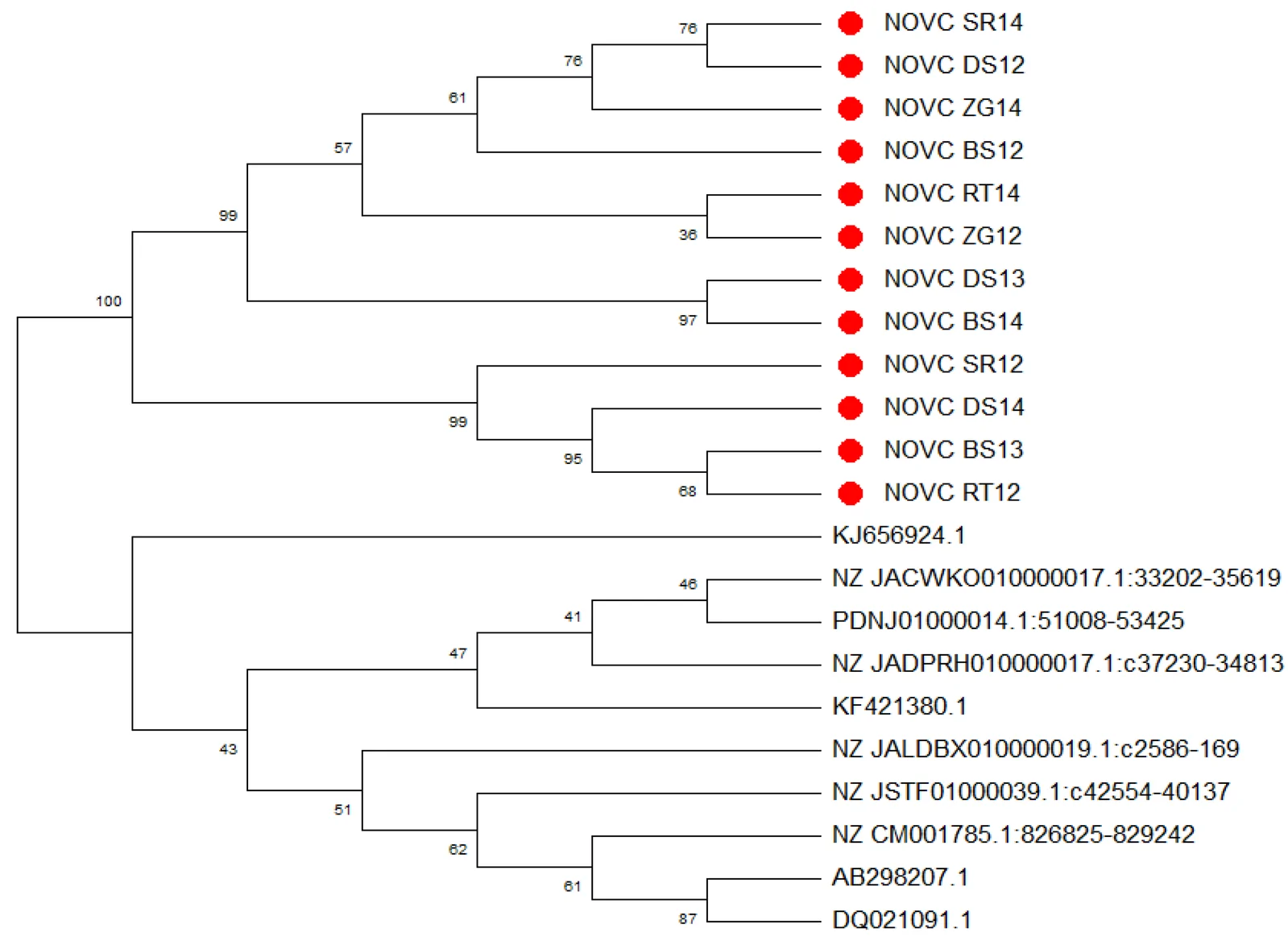
Neighbour-joining phylogenetic tree constructed using MEGA v12.0. The tree is based on concatenated nucleotide sequences of the gyrB gene from NOVC isolates recovered from wastewater and ten reference sequences obtained from NCBI. Sequences of wastewater isolates are highlighted in dots. Alignments were performed using ClustalW, and bootstrap values at the internodes, calculated from 1000 replicates, indicate the robustness of each cluster.

To gain a global perspective on the genetic relationships of NOVC isolates, a neighbour-joining phylogenetic tree was constructed based on the gyrB gene. Reference genomes were selected from diverse regions including South Africa, the Netherlands, China, the USA, Ghana, Russia, India, Japan, France, and Australia to ensure broad representation. The resulting phylogeny revealed notable genetic diversity among the wastewater-derived NOVC isolates and reference genomes, which clustered into two distinct groups relative to the global strains. This pattern highlights both local and global genetic relationships and suggests the presence of genetically divergent lineages circulating in the Tshwane district wastewater treatment plants, potentially reflecting multiple sources or evolutionary pathways. The relationship between the NOVC isolates and the reference genome is shown in Figure 6.

## 4. Discussion

Wastewater-based epidemiology is an important tool for monitoring the transmission of *V. cholerae* in communities [21]. Several studies have detected both the toxigenic and non-toxigenic strains in wastewater samples [21,22]. The epidemiological significance of environmental *V. cholerae* strains remains unclear, as most strains recovered from environmental sources do not produce cholera toxin and lack the virulence gene cassette [6]. Strains of the non-O1/non-O139 serogroups are indigenous in the marine, estuarine, and inland water environments. This study aimed to characterise NOVC isolates in wastewater in the Tshwane district. Of the 341 wastewater samples analysed, 319 yielded *V. cholerae* isolates of which 143 were identified as NOVC by PCR. A similar study was conducted by Baron et al. (2017), which reported the presence of NOVC in 75% of wastewater samples in comparison to other samples used in the study [21].

The emergence of antimicrobial resistance in NOVC strains is reported with increasing frequency, particularly in regions affected by recurrent cholera outbreaks [23]. The consequent decline in the effectiveness of widely accessible antibiotics poses a serious risk to public health [23]. In this study, antimicrobial susceptibility profiles of NOVC isolates circulating in the Tshwane district were assessed; according to phenotypic AST results, these isolates displayed high sensitivity to most of the first-line drugs such as trimethoprim–sulfamethoxazole, levofloxacin, and ampicillin, suggesting that these remain effective treatment options for the treatment of infections caused by NOVC isolates. The high susceptibility rates observed in this study may be attributed to less exposure of NOVC to antibiotics, as these strains are rarely reported in clinical settings because they predominantly persist in environmental habitats [24]. Almost half (46%) of the isolates exhibited resistance to meropenem, an unexpected finding given that this antibiotic is not routinely used in the treatment of *V. cholerae* infections. A study conducted by Ottaviani et al. (2009) reported similar findings, which showed high susceptibility rates in most of the drugs used for treatment such as trimethoprim–sulfamethoxazole, chloramphenicol, ciprofloxacin and cefotaxime, also showing intermediate resistance to meropenem [25]. Wastewater is regarded as a hotspot for the development of antimicrobial resistance [21]. Nevertheless, in this study, resistance was quite low and observed in one antibiotic.

Whole genome sequencing (WGS) is an important technique used to investigate and characterise information carried by the genome of bacterial pathogens, such as genetic relatedness between different isolates and organisms, the mechanism of antimicrobial resistance (AMR) in bacterial organisms and virulence factors [22]. A total of twelve isolates were fully sequenced, and ten isolates were identified as *V. cholerae* while two were identified as *Vibrio* species. The sequence types of these isolates were not identified, and this could be attributed to limitations in existing MLST databases or the presence of novel allelic profiles not yet represented in current MLST schemes, a scenario commonly observed among diverse NOVC populations [26,27]. In this study, WGS combined with comparative genomics offers valuable insights into the genetic diversity of the NOVC isolates from wastewater. The genomic features of the NOVC reported in this study, such as genome size, total number of CDSs, tRNA, rRNA, tmRNA, sORF, CRISPR elements, and GC content, are similar to those of other NOVC strains [4].

Whole genome sequencing further revealed that none of the isolates harboured toxigenic genes associated with virulence, confirming their classification as NOVC. The *ctxA* and *tcpA* genes, traditionally associated with cholera epidemics and pandemics, are usually absent from these strains [28]. Similar findings were documented in a study conducted by Mkhize et al. (2025) in Johannesburg, South Africa, where a total of 24 isolates lacked *ctxA* and *tcpA* and were subsequently identified as NOVC isolates [29]. Several other studies have found that some NOVC strains lack important virulence genes such as *ctxA* and *tcpA* [30,31]. However, these environmental isolates may still possess other virulence factors contributing to their pathogenicity [32,33]. The isolates analysed in this study were found to harbour several other virulence-associated genes, including *hlyA*, *rtxA*, *ompU*, *gyrB*, *gyrA*, *trkH*, and *tnaA*, which are implicated in various infection-related processes and may contribute to the pathogenic potential of these strains. *V. cholerae* contains a large amount of *hlyA*, which has a unique structural domain that binds to target cells and may be a major factor in NOVC’s capacity to enter the bloodstream [32].

The genetic antibiotic resistance profiles of the ten NOVC isolates in this study were characterised using CARD and ResFinder (EPI2ME). In silico analysis identified multiple resistance genes, which confer resistance to a broad range of antimicrobial agents including the first-line drugs such as trimethoprim–sulfamethoxazole, ampicillin, and ciprofloxacin. A study conducted in China by Tang et al. (2023) also revealed the presence of *CRP*, *varG*, *almG*, and *qnrVC4* genes conferring resistance to multiple antibiotics [33]. The isolates also consisted of regulatory genes (*CRP*, *rsmA* and *almF*) involved in controlling efflux pump genes and systems which play a significant role in the resistance of *V. cholerae* against antibiotics [24]. Furthermore, these isolates also carried genes conferring resistance to fluoroquinolones (*parE* and *qnrVC4*), aminoglycosides (*aph (6)-Id* and *aph(3*″*)-Ib*), polymyxins (*almF*), glycopeptides (*vanT* in the *vanG* cluster), trimethoprim–sulfamethoxazole (*dfrA31*), and β-lactams (*CARB-9*). The differences in the phenotypic and genome-based antimicrobial resistance assessments indicate that existing resistance databases are currently inadequate for evaluating antimicrobial resistance in NOVC strains [24]. These discrepancies arise from substantial global variation in resistance trends and patterns among NOVC [21]. In addition, limited information is available on the genetic and resistance determinants of these strains, as they were previously overlooked. Continuous monitoring and comparison of phenotypic and genotypic data may help to resolve these discrepancies and provide additional data to the resistance database for accurate evaluation [24]. *V. cholerae* typically acquires antimicrobial resistance through mechanisms such as target replacement, target protection, and enzymatic inactivation through hydrolysis [34]. This prevents access to the target site by altering membrane permeability and actively exporting antibiotics from cells [34,35]. Mobile genetic elements, and plasmids in particular, play a crucial role in the spread of antibiotic resistance [36]. The analysis of NOVC isolates using mobileOG-db revealed various MGEs across the study isolates. The presence of phages, replication/recombination/repair, stability/transfer/defence, integration/excision and transfer functional groups enables these isolates to contribute to disease development, AMR, and phage infection and facilitate HGT [16]. A high prevalence of replication, recombination, and repair (RRR) genes, as well as phage-associated elements, was observed across the isolates. These functional modules are commonly associated with plasmids and phages, which often carry RRR proteins that have close homologs in chromosomal DNA [35].

Plasmids have been implicated in HGT and contribute significantly to the evolution of microorganisms [37,38]. In this study, several plasmids were identified within contigs carrying several resistance, virulence, and toxin antitoxin genes. Sequence analysis revealed that the plasmids encoded a range of functional elements, including resistance determinants, relaxase, replicase, metabolic, hydrolase and transport membrane genes, toxin–antitoxin systems, and hypothetical proteins, indicating the dissemination of antimicrobial resistance and virulence traits. Resistance genes such as *dfrA31*, *dfrA6*, *dfrA1 QNR_VIBPA*, *dhfrI*, *qnrVC* and *qnrVC5* were found exclusively in plasmids belonging to three distinct isolates: RT14 (contig six), BS12 (contig 9 and 10) and BS14 (contig 94). These genes are known to confer resistance to trimethoprim and quinolones. Plasmids of different enteric pathogens, including *V. cholerae*, have been reported to have several physically connected genetic determinants that confer resistance to various antibiotic classes [23].

The replication initiation protein (rep) was identified in a plasmid from one isolate, BS13 (contig 42). This protein is essential for plasmid replication and maintenance and is often used as a marker for plasmid compatibility [39]. A pseudoknot structure of the regulatory region of the *repBA* gene was also detected in the same isolate. This structure plays an important role in controlling plasmid replication by regulating translation of the *repA* replication initiator protein and influencing gene expression at the RNA level [40]. All plasmids carried hypothetical proteins, which are uncharacterised since their function is unknown and no close homologs could be identified [41]. Mobilisable plasmids typically possess a mobilisation (mob) region that encodes specific components of the relaxosome and the origin of transfer (*oriT*) [42]. Plasmids carrying genes encoding relaxase enzymes were identified in several isolates, including BS13, BS14 and SR12. Relaxases are essential components of the relaxosome, responsible for initiating DNA transfer in both conjugative and mobilisable plasmids [43]. The *MobM* protein was found in two plasmids from isolates BS12 (contig 1) and BS14 (contig 94). This protein encodes a relaxase enzyme, which plays a key role in conjugative transfer [44]. Additionally, *MobA* and *MobB* proteins were identified in a plasmid from isolate BS13 (contig 42), while *MobC* was also detected in a plasmid belonging to isolate BS13 (contig 42). *MobC* forms the relaxosome complex by interacting with *MobA* and *MobB* [45]. To facilitate DNA transfer, *MobA* proteins bind and nick double-stranded DNA at the origin of transfer (*oriT*) site [44]. *MobB* is also essential for efficient mobilisation, as it stabilises the relaxosome and increases the proportion of plasmid molecules nicked at *oriT* [46].

*MbeA* and *MbeC* proteins were also identified in a plasmid from isolate BS13 (contig 42). The *MbeA* protein is essential for plasmid mobilisation, while *MbeC* plays a key role in facilitating the specific transfer of the plasmid during conjugation [44]. Detection of relaxase/mobilisation nuclease domain-containing protein in a plasmid from BS13 (contig 42) supports its potential role in HGT [47]. These plasmids are classified as mobilisable plasmids due to the presence of relaxase genes. The presence of multiple relaxase enzymes may affect various aspects of the bacterial life cycle beyond their role in horizontal gene transfer [48]. Relaxases are not limited to initiating conjugative transfer from their corresponding *oriT* sites; they can also participate in additional cellular processes that affect plasmid maintenance, DNA processing, and host adaptation [48].

The *TraY* protein was identified in plasmids from isolates RT14 (contig 6) and is an important factor for DNA transfer, typically found in conjugative plasmids [49]. Plasmids belonging to isolates BS14 (contig 94) and RT14 (contig 06) carried toxin–antitoxin genes (*parD1*, *parD*, *parE*, *toxin*, *toxin–antitoxin system*, *antitoxin component*, and *ribbon–helix–helix domain protein*), which act as plasmid stabilisation systems. These genes help to ensure plasmid survival by supporting DNA replication and translation and by giving bacteria tolerance to antibiotics, especially under stressful conditions [44].

Plasmids are capable of acquiring genes from various organisms through HGT, a mechanism that plays a crucial role in bacterial evolution, enabling genetic adaptation and transmission of traits such as stress tolerance and antibiotic resistance [50]. In this study, several proteins often associated with other organisms were identified in plasmids within various isolates. For example, the *CppC* protein was detected in the plasmid of isolate BS13 (contig 42); this protein has previously been associated with *Neisseria gonorrhoeae* [45]. Additional genes, including *udg*, *ugd*, *eco47IIM*, *haeIIIM*, *ybbA*, *lolD*, *ESTE_VIBMI*, *atpF*, *atpE*, *atpB*, *atpA*, *mnmE*, *parB*, *mioC*, *mnmG*, *rsmG*, *soJ*, and *fabV*, were found to share similarity with sequences from different organisms such as *Escherichia coli*, *Salmonella enterica*, *Salmonella typhimurium*, *V. cholerae* O1 serotype, *Haemophilus influenzae*, *Psychromonas ingrahamii*, *Idiomarina loihiensis*, *Coxiella burnetii*, *Vibrio mimicus*, *Bacillus subtilis*, and *Clostridium acetobutylicum*. These genes play a significant role in the adaptability, metabolic flexibility, and plasmid partitioning of the organism [51]. L-asparaginase and *CbiA* were also detected in isolates BS14 (contig 94) and RT14 (contig 6). These enzymes contribute to the organism’s ability to adapt to nitrogen-limited environments, thereby enhancing its metabolic flexibility and ecological fitness [52].

All plasmids in this study were smaller than 50 kb, which explains why they carried only a few resistance genes. This is consistent with earlier studies which showed that multidrug-resistant plasmids are usually larger than 50 kb, are self-conjugative, and have more complex systems to control their copy number and replication [44]. Interestingly, all plasmids lacked the usual origin of replication, except a plasmid belonging to isolate BS14 (contig 19). Reports suggest that such plasmids may use other replication mechanisms. For example, other plasmids have been shown to replicate using the ColE1 model of replication [38]. These findings highlight the various plasmids found, their significant role in drug resistance, virulence, and toxin acquisition, and their overall contribution to the adaptive biology of *V. cholerae*.

## 5. Conclusions

Wastewater-based epidemiology is a valuable approach for early detection of infectious diseases within communities. In this study, WGS was used to investigate virulence and antimicrobial resistance in NOVC isolates from wastewater. Although these isolates did not carry the toxigenic genes, they harboured alternative virulence factors that may contribute to pathogenicity and pose a potential risk for clinical infection. Resistance to several first-line antibiotics was also observed, underscoring the urgent need for continuous surveillance to prevent the emergence of these strains as a significant public health threat.

## Figures and Tables

**Table 1:** Bacterial isolate characterisation of NOVC isolates, including genome size and contig information.

Isolate ID	Taxon	Genome Size
BS12	*V. cholerae*	3.87 Mb
BS13	*V. cholerae*	4.25 Mb
BS14	*V. cholerae*	3.73 Mb
DS14	*V. cholerae*	3.95 Mb
ZG12	*V. cholerae*	4.13 Mb
ZG14	*V. cholerae*	4.92 Mb
RT12	*V. cholerae*	3.95 Mb
RT14	*V. cholerae*	3.86 Mb
SR12	*V. cholerae*	3.84 Mb
SR14	*V. cholerae*	3.86 Mb

**Table 2:** Genomic features of NOVC isolates recovered from wastewater treatment plants in the Tshwane district.

Isolate ID	G + C Content (%)	Genes	CDS	tRNA	tmRNA	rRNA	sORF	CRISPR
BS12	48%	6459	6328	96	1	33	6800	1
BS13	47%	7152	7031	90	1	29	7273	1
BS14	48%	7474	7365	88	0	20	6180	1
DS14	48%	4289	4157	102	1	28	5983	1
ZG12	47%	3761	3624	100	1	32	5221	1
ZG14	48%	8446	8302	109	1	34	8727	0
RT12	48%	3562	3430	102	1	29	5385	0
RT14	47%	5154	5022	100	1	29	6297	2
SR12	47%	3564	3434	101	1	27	5221	1
SR14	44%	4527	4397	98	1	27	4672	4

Guanine + cytosine content (G + C content, %), genes, coding DNA sequences (CDS), transfer RNA (tRNA), transfer-messenger RNA (tmRNA), ribosomal RNA (rRNA), small open reading frames (sORFs), clustered regularly interspaced short palindromic repeats (CRISPR).

**Table 3:** Prevalence and distribution of functional categories in NOVC isolates.

Isolate ID	P	IE	RRR	STD	T	Total
BS12	69	31	118	24	29	271
BS13	41	112	119	24	30	326
BS14	74	29	122	19	22	266
DS14	33	45	77	47	21	223
ZG12	30	65	69	26	15	205
ZG14	127	54	125	25	37	378
RT12	27	29	70	21	12	159
RT14	44	43	100	11	28	226
SR12	29	26	66	15	16	152
SR14	32	152	99	44	51	378

P (phage), integration/excision (IE), replication/recombination/repair (RRR), stability/transfer/defence (STD), and transfer (T).

**Table 4:** Summary of antimicrobial resistance profiles of NOVC isolates, showing predicted resistance genes, associated mechanisms, drug classes, and specific antimicrobial agents.

*V. cholerae* Isolates	Genes	Antibiotics	Drug Classes	Resistance Mechanisms
BS12	*qnrVC4*	CIP	FQ	Target protection
BS13	*BlaCARB-9*	AMP, AMO, PIP	PEN	Enzyme degradation
BS14	*qnrVC5*, *dfrA31*	CIP, TMP	FQ, FPI	Antibiotic target protection and target replacement
DS14	*aph(6)-id*, *aph(3*″*)-Ib*, *floR*, *dfrA1*	SMX, TMP, STR, CHL, FFC,	AMG, PH, FPI	Antibiotic inactivation, antibiotic efflux, and antibiotic replacement
ZG12	*BlaCARB-9*	AMP, AMO, PIP	PEN	Enzyme degradation
ZG14	*blaDHA-9*, *blaDHA-19*, *BlaCARB-7*, *dfrA1*	AMP, CTX, CAZ, PIP, AMO	CEP, PEN	Enzymatic degradation and target replacement
RT12	*qnrVC4*, *dfrA31*	CIP, AMP, AMO, PIP, TMP	PEN, FQ, FPI	Efflux pump, enzymatic inactivation, and target replacement
RT14	*qnrVC5*, *dfrA31*	CIP, TMP	FQ, FPI	Antibiotic target protection and target replacement
SR12	*qnrVC4*	CIP	FQ	Target protection
SR14	*dfrA6*, *ere(A)*	TMP, ERY,	FPI, MAC	Target replacement

**Table 5:** Distribution of virulence-associated genes in NOVC isolates, categorised by functional groups including cytolytic factors, adhesion and colonisation factors, secretion systems, biofilm-associated genes, and regulatory/metabolic elements.

	Virulence Factors												
Isolates	Toxins and Cytolytic Factors			Adhesion and Colonisation					Secretion Systems				
	*hlyA*	*rtxA*	*stn*	*mshA*	*ompU*	*tcpA*	*HA/P*	*nagH*	*T3SS*	*T6SS*	*RtxC*	*VPI-1*	*VPI-2*
BS12	+	+	-	+	+	-	-	-	-	-	-	-	-
BS13	+	+	-	+	+	-	-	-	-	-	-	-	-
DS14	+	+	-	+	+	-	-	-	-	-	-	-	-
BS14	+	+	-	-	+	-	-	-	-	-	-	-	-
ZG14	+	+	-	+	+	-	-	-	-	-	-	-	-
RT14	+	-	-	+	+	-	-	-	-	-	-	-	-
SR14	+	-	-	-	-	-	-	-	-	-	-	-	-
ZG12	+	-	-	+	+	-	-	-	-	-	-	-	-
RT12	+	-	-	+	+	-	-	-	-	-	-	-	-
SR12	+	-	-	+	+	-	-	-	-	-	-	-	-
	**Virulence Factors**												
**Isolates**	**Biofilm**			**Regulatory and Metabolic**									
	***VSP1/VSP2***	***als***	***makA***	***toxR***	***VSPR***	***rfbv***	***rfbc***	***trkH***	***tnaA***	***gyrA***	***gyrB***		
BS12	-	-	-	-	-	-	-	+	+	+	+		
BS13	-	-	-	-	-	-	-	+	+	+	+		
DS14	-	-	-	-	-	-	-	+	+	+	+		
BS14	-	-	-	-	-	-	+	+	+	+	+		
ZG14	-	-	+	-	+	-	-	+	+	-	+		
RT14	-	-	-	-	-	-	-	+	+	+	+		
SR14	-	-	-	-	-	-	-	+	+	+	+		
ZG12	-	-	-	-	-	-	-	+	+	+	+		
RT12	-	-	-	-	-	-	+	+	+	+	+		
SR12	-	-	-	-	-	-	-	+	+	+	+		

## Data Availability

The original contributions presented in this study are included in the article/Supplementary Materials. Further inquiries can be directed to the corresponding author.

## Supplementary Materials

The following supporting information can be downloaded at: https://www.mdpi.com/article/10.3390/microorganisms14091995/s1, Table S1: Primers used for amplification by PCR; Table S2: Primers used for rep-PCR; Table S3: Comparative analysis of various plasmids detected in NOVC isolates; Table S4: Summary of antimicrobial resistance profiles of NOVC isolates, showing predicted resistance genes, associated mechanisms, drug classes, and specific antimicrobial agents; Table S5: Distribution of virulence-associated genes in non-O1/non-O139 *Vibrio cholerae* isolates, categorised by functional groups including cytolytic factors, adhesion and colonization factors, secretion systems, biofilm-associated genes, and regulatory/metabolic element; Supplementary S1: Sequencing depth/coverage; Supplementary S2: Plasmids; Supplementary S3: Whole genome maps.

## References

[1] Montero D.A., Vidal R.M., Velasco J., George S., Lucero Y., Gómez L.A., Carreño L.J., García-Betancourt R., O’Ryan M. (2023). *Vibrio cholerae*, classification, pathogenesis, immune response, and trends in vaccine development. Front. Med..

[2] Ortega D.R., Kjær A., Briegel A. (2020). The chemosensory systems of *Vibrio cholerae*. Mol. Microbiol..

[3] Liang K.Y., Orata F.D., Islam M.T., Nasreen T., Alam M., Tarr C.L., Boucher Y.F. (2020). A *Vibrio cholerae* Core Genome Multilocus Sequence Typing Scheme to Facilitate the Epidemiological Study of Cholera. J. Bacteriol..

[4] Wang H., Yang C., Sun Z., Zheng W., Zhang W., Yu H., Wu Y., Didelot X., Yang R., Pan J. (2020). Genomic epidemiology of *Vibrio cholerae* reveals the regional and global spread of two epidemic non-toxigenic lineages. PLoS Negl. Trop. Dis..

[5] Bhandari M., Jennison A.V., Rathnayake I.U., Huygens F. (2021). Evolution, distribution and genetics of atypical *Vibrio cholerae*—A review. Infect. Genet. Evol..

[6] Singh D.V., Matte M.H., Matté G.R., Jiang S., Sabeena F., Shukla B.N., Sanyal S.C., Huq A., Colwell R. (2001). Molecular Analysis of *Vibrio cholerae*O1, O139, non-O1, and non-O139 Strains: Clonal Relationships between Clinical and Environmental Isolates. Appl. Environ. Microbiol..

[7] Fu S., Hao J., Jin S., Wu K., Wang Y., Ye S., Liu Y., Li R. (2019). A Human Intestinal Infection Caused by a Novel Non-O1/O139 *Vibrio cholerae* Genotype and Its Dissemination Along the River. Front. Public Health.

[8] Vezzulli L., Baker-Austin C., Kirschner A., Pruzzo C., Martinez-Urtaza J. (2020). Global emergence of environmental non-O1/O139 *Vibrio cholerae* infections linked with climate change: A neglected research field?. Environ. Microbiol..

[9] Li X., Wu Y., Sun X., Ma J., Li X., Liu C., Xie H. (2020). Non-O1/non-O139 *Vibrio cholerae* bacteraemia in mainland China from 2005 to 2019: Clinical, epidemiological and genetic characteristics. Epidemiol. Infect..

[10] Huq A., Haley B.J., Taviani E., Chen A., Hasan N.A., Colwell R.R. (2012). Detection, Isolation, and Identification of *Vibrio cholerae* from the Environment. Curr. Protoc. Microbiol..

[11] Le Roux F., Davis B.M., Waldor M.K. (2011). Conserved small RNAs govern replication and incompatibility of a diverse new plasmid family from marine bacteria. Nucleic Acids Res..

[12] Nandi B., Nandy R.K., Mukhopadhyay S., Nair G.B., Shimada T., Ghose A.C. (2000). Rapid Method for Species-Specific Identification of *Vibrio cholerae* Using Primers Targeted to the Gene of Outer Membrane Protein OmpW. J. Clin. Microbiol..

[13] Kolmogorov M., Yuan J., Lin Y., Pevzner P.A. (2019). Assembly of long, error-prone reads using repeat graphs. Nat. Biotechnol..

[14] Bortolaia V., Kaas R.S., Ruppe E., Roberts M.C., Schwarz S., Cattoir V., Philippon A., Allesoe R.L., Rebelo A.R., Florensa A.F. (2020). ResFinder 4.0 for predictions of phenotypes from genotypes. J. Antimicrob. Chemother..

[15] Alcock B.P., Huynh W., Chalil R., Smith K.W., Raphenya A.R., Wlodarski M.A., Edalatmand A., Petkau A., Syed S.A., Tsang K.K. (2023). CARD 2023: Expanded curation, support for machine learning, and resistome prediction at the Comprehensive Antibiotic Resistance Database. Nucleic Acids Res..

[16] Brown C.L., Mullet J., Hindi F., Stoll J.E., Gupta S., Choi M., Keenum I., Vikesland P., Pruden A., Zhang L. (2022). mobileOG-db: A Manually Curated Database of Protein Families Mediating the Life Cycle of Bacterial Mobile Genetic Elements. Appl. Environ. Microbiol..

[17] Saitou N., Nei M. (1987). The neighbor-joining method: A new method for reconstructing phylogenetic trees. Mol. Biol. Evol..

[18] Felsenstein J. (1985). Phylogenies and the comparative method. Am. Nat..

[19] Tamura K., Nei M., Kumar S. (2004). Prospects for inferring very large phylogenies by using the neighbor-joining method. Proc. Natl. Acad. Sci. USA.

[20] Kumar S., Stecher G., Suleski M., Sanderford M., Sharma S., Tamura K. (2024). MEGA12: Molecular evolutionary genetic analysis version 12 for adaptive and green computing. Mol. Biol. Evol..

[21] Baron S., Larvor E., Chevalier S., Jouy E., Kempf I., Granier S.A., Lesne J. (2017). Antimicrobial Susceptibility among Urban Wastewater and Wild Shellfish Isolates of Non-O1/Non-O139 *Vibrio cholerae* from La Rance Estuary (Brittany, France). Front. Microbiol..

[22] Uelze L., Grützke J., Borowiak M., Hammerl J.A., Juraschek K., Deneke C., Tausch S.H., Malorny B. (2020). Typing methods based on whole genome sequencing data. One Health Outlook.

[23] Dutta D., Kaushik A., Kumar D., Bag S. (2021). Foodborne Pathogenic Vibrios: Antimicrobial Resistance. Front. Microbiol..

[24] Lepuschitz S., Baron S., Larvor E., Granier S.A., Pretzer C., Mach R.L., Farnleitner A.H., Ruppitsch W., Pleininger S., Indra A. (2019). Phenotypic and Genotypic Antimicrobial Resistance Traits of *Vibrio cholerae* Non-O1/Non-O139 Isolated from a Large Austrian Lake Frequently Associated with Cases of Human Infection. Front. Microbiol..

[25] Ottaviani D., Leoni F., Rocchegiani E., Santarelli S., Masini L., Di Trani V., Canonico C., Pianetti A., Tega L., Carraturo A. (2009). Prevalence and virulence properties of non-O1 non-O139 *Vibrio cholerae* strains from seafood and clinical samples collected in Italy. Int. J. Food Microbiol..

[26] Octavia S., Salim A., Kurniawan J., Lam C., Leung Q., Ahsan S., Reeves P.R., Nair G.B., Lan R. (2013). Population Structure and Evolution of Non-O1/Non-O139 *Vibrio cholerae* by Multilocus Sequence Typing. PLoS ONE.

[27] Arteaga M., Velasco J., Rodriguez S., Vidal M., Arellano C., Silva F., Carreño L.J., Vidal R., Montero D.A. (2020). Genomic characterization of the non-O1/non-O139 *Vibrio cholerae* strain that caused a gastroenteritis outbreak in Santiago, Chile, 2018. Microb. Genom..

[28] Luo Y., Ye J., Jin D., Ding G., Zhang Z., Mei L., Octavia S., Lan R. (2013). Molecular analysis of non-O1/non-O139 *Vibrio cholerae* isolated from hospitalised patients in China. BMC Microbiol..

[29] Mkhize L., Marimani M., Duze S.T. (2025). Characterization of *Vibrio cholerae* from the Jukskei River in Johannesburg, South Africa. Lett. Appl. Microbiol..

[30] Iyer L., Vadivelu J., Puthucheary S.D. (2000). Detection of virulence associated genes, haemolysin and protease amongst *Vibrio cholerae* isolated in Malaysia. Epidemiol. Infect..

[31] Rajpara N., Vinothkumar K., Mohanty P., Singh A.K., Singh R., Sinha R., Nag D., Koley H., Kushwaha Bhardwaj A. (2013). Synergistic Effect of Various Virulence Factors Leading to High Toxicity of Environmental *V. cholerae* Non-O1/ Non-O139 Isolates Lacking *ctx* Gene: Comparative Study with Clinical Strains. PLoS ONE.

[32] Yang W., Li P., Lei S., Yu Y., Liu S., You C. (2024). Case report: Characterization and bioinformatics analysis of non-O1/O139 *Vibrio cholerae* strain isolated from a choledochoduodenal fistula patient with septicemia. Front. Med..

[33] Tang J., Li S., Zhang M., Li F., Tang Y., Yang F. (2023). Whole Genome Analysis of a Non-O1, Non-O139 *Vibrio cholerae* Detected from Human Blood in China. Infect. Drug Resist..

[34] Mevada V., Patel R., Dudhagara P., Chaudhari R., Vohra M., Khan V., JHShyu D., Chen Y.Y., Zala D. (2023). Whole genome sequencing and pan-genomic analysis of multidrug-resistant *Vibrio cholerae* VC01 isolated from a clinical sample. Microorganisms.

[35] Chen Y.T., Tang H.J., Chao C.M., Lai C.C. (2015). Clinical Manifestations of Non-O1 *Vibrio cholerae* Infections. PLoS ONE.

[36] MacLean R.C., San Millan A. (2015). Microbial Evolution: Towards Resolving the Plasmid Paradox. Curr. Biol..

[37] Carattoli A. (2013). Plasmids and the spread of resistance. Int. J. Med. Microbiol..

[38] Ceccarelli D., Garriss G., Choi S.Y., Hasan N.A., Stepanauskas R., Pop M., Huq A., Colwell R.R. (2017). Characterization of Two Cryptic Plasmids Isolated in Haiti from Clinical *Vibrio cholerae* Non-O1/Non-O139. Front. Microbiol..

[39] Zatyka M., Thomas C.M. (1998). Control of genes for conjugative transfer of plasmids and other mobile elements. FEMS Microbiol. Rev..

[40] Praszkier J., Pittard A.J. (2005). Control of replication in I-complex plasmids. Plasmid.

[41] Díaz-Orejas R., Espinosa M., Yeo C.C. (2017). The importance of the expendable: Toxin–antitoxin genes in plasmids and chromosomes. Front. Microbiol..

[42] Rawlings D.E., Tietze E. (2001). Comparative biology of IncQ and IncQ-like plasmids. Microbiol. Mol. Biol. Rev..

[43] Piergentili P., Castellani-Pastoris M.A., Fellini R.D., Farisano G., Bonello C., Rigoli E., Zampieri A. (1984). Transmission of Non O Group 1 *Vibrio cholerae* by Raw Oyster Consumption. Int. J. Epidemiol..

[44] Francia M.V., Varsaki A., Garcillán-Barcia M.P., Latorre A., Drainas C., de la Cruz F. (2004). A classification scheme for mobilization regions of bacterial plasmids. FEMS Microbiol. Rev..

[45] Korch C., Hagblom P., Ohman H., Göransson M., Normark S. (1985). Cryptic plasmid of Neisseria gonorrhoeae: Complete nucleotide sequence and genetic organization. J. Bacteriol..

[46] Perwez T., Meyer R. (1996). MobB protein stimulates nicking at the R1162 origin of transfer by increasing the proportion of complexed plasmid DNA. J. Bacteriol..

[47] Iyer L.M., Burroughs A.M., Anand S., de Souza R.F., Aravind L. (2017). Polyvalent proteins, a pervasive theme in the intergenomic biological conflicts of bacteriophages and conjugative elements. J. Bacteriol..

[48] Guzmán-Herrador D.L., Llosa M. (2019). The secret life of conjugative relaxases. Plasmid.

[49] Vollmer W., von Rechenberg M., Höltje J.V. (1999). Demonstration of Molecular Interactions between the Murein Polymerase PBP1B, the Lytic Transglycosylase MltA, and the Scaffolding Protein MipA of *Escherichia coli**. J. Biol. Chem..

[50] Harrison E., Brockhurst M.A. (2012). Plasmid-mediated horizontal gene transfer is a coevolutionary process. Trends Microbiol..

[51] Hasan N.A., Grim C.J., Haley B.J., Chun J., Alam M., Taviani E., Hoq M., Munk A.C., Saunders E., Brettin T.S. (2010). Comparative genomics of clinical and environmental *Vibrio mimicus*. Proc. Natl. Acad. Sci. USA.

[52] Srikhanta Y.N., Atack J.M., Beacham I.R., Jennings M.P. (2013). Distinct physiological roles for the two l-asparaginase isozymes of *Escherichia coli*. Biochem. Biophys. Res. Commun..

