# Supplementary material for: Whole Genome Characterisation of Non-O1/Non-O139 Vibrio cholerae in Wastewater Treatment Plants in the Tshwane District, South Africa
Source: Microorganisms. 2026 Sep 9;14(9):1995. doi: 10.3390/microorganisms14091995 (PMC13609860; doi:10.3390/microorganisms14091995)
Supplement: Supplementary file 1 [file microorganisms-14-01995-s001.zip › microorganisms-4305776-supplementary.pdf]

Table S1: Primers used for amplification by PCR

Target gene	Primer Sequence (5'-3')	Amplicon size (bp)	PCR Conditions	Reference
ompW	F: CACCAAGAAGGTGACTTTATTGTG	304	Initial denaturation at 94 °C for 4 minutes, 35 cycles of denaturation at 94 °C for 1 minute, annealing at 50 °C for 1 minute, and extension at 72 °C for 5 minutes; and final extension at 72 °C for 5 minutes.	(Goel *et al*, 2007)
	R: GAACTTATAACCACCCGCG			
rfb-O1	F: GACTGTCAGCTGGCGGAA	192	Initial denaturation at 94 °C for 5 minutes, 35 cycles of denaturation at 94 °C for 1 minute, annealing at 55 °C for 1 minute, an extension at 72 °C for 1 minute and final extension at 72 °C for 7 minutes.	(Bhumiratana *et al*, 2014)
	R: GTT GGC GTA TTA CGG TAC			
rfb-O139	F: GCGTTTATCGCCGGTCGAC	449	Initial denaturation at 94 °C for 5 minutes, 35 cycles of denaturation at 94 °C for 1 minute, annealing at 55 °C for 1 minute, an extension at 72 °C for 1 minute and final extension at 72 °C for 7 minutes.	(Bhumiratana *et al*, 2014)
	R: GTAACTTGGTACAATCTCG			
tcpA	F: CACGATAAGAAAACCGGTCAAGAG	295	Initial denaturation at 94 °C for 4 minutes,	Ceccarelli *et al.*, 2015

	R: TTACCAAATGCAACGCCGAATG		35 cycles of denaturation at 94 °C for 1 minute, annealing at 50 °C for 1 minute, and extension at 72 °C for 5 minutes; and final extension at 72 °C for 5 minutes.	
ctxA	F: CGGGCAGATTCTAGACCTCCTG	219	Initial denaturation at 94 °C for 4 minutes, 35 cycles of denaturation at 94 °C for 1 minute, annealing at 50 °C for 1 minute, and extension at 72 °C for 5 minutes; and final extension at 72 °C for 5 minutes.	Daboul *et al.*, 2020
	R: CGATGATCTTGGAGCATTCCCAC			

Table S2: Primers used for rep-PCR

Primer	Primer sequence	PCR conditions	References
Rep-PCR	F:5' - ICGICTTATCIGGCCTA-3'	Initial denaturation at 94°C for 10 minutes, 35 cycles of denaturation at 94°C for 1 minute, annealing at 45°C for 1 minute, and extension at 72°C for 2 minutes; and final extension at 72°C for 16 minutes.	(Ramazanzadeh *et al.*, 2015)
	R: 5' – IIIICGICATCIGGC-3'		

Supplementary S1: Sequencing depth/coverage

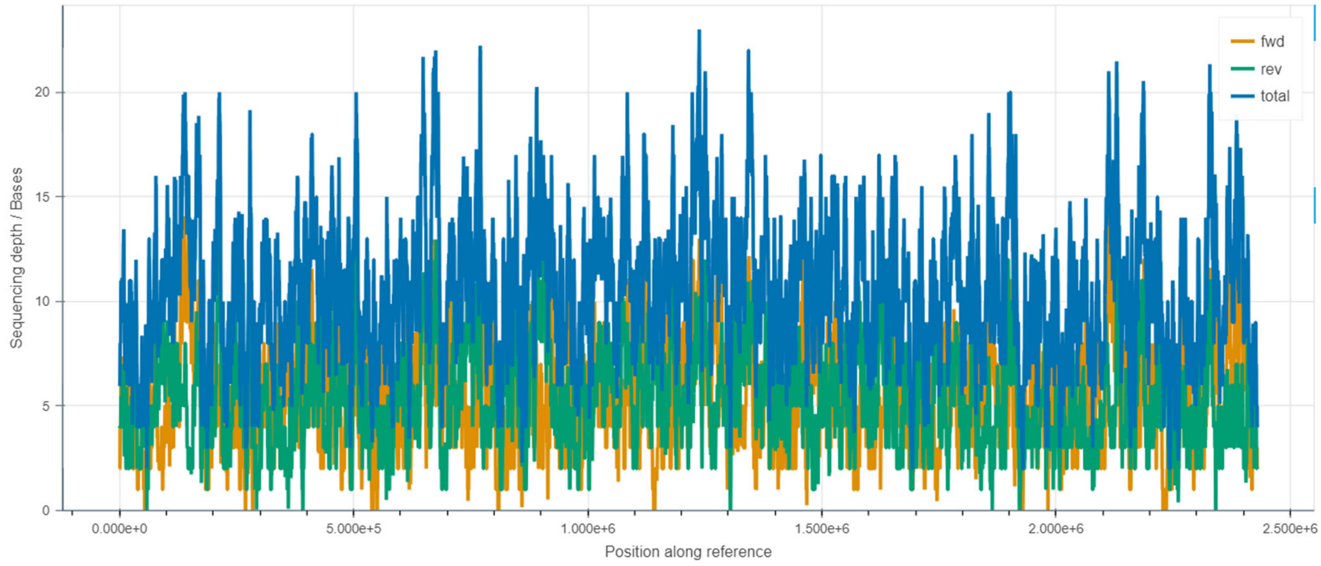

Supplementary S2: Plasmids

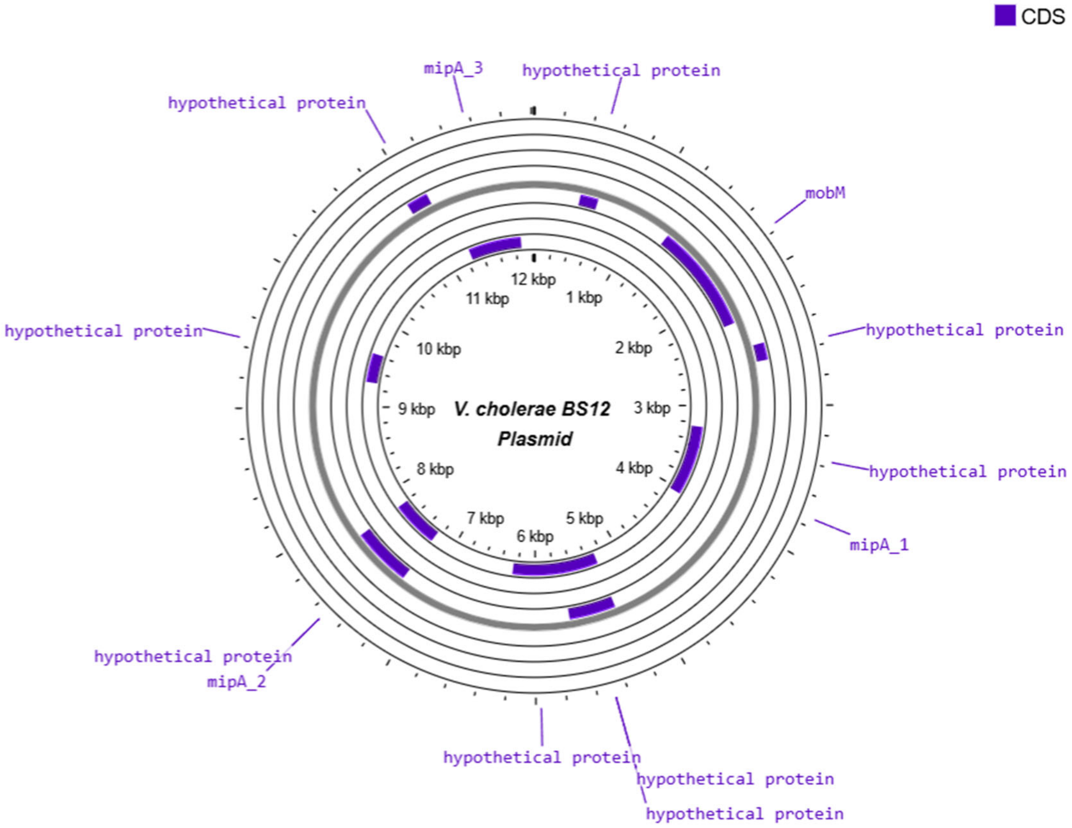

Figure S1: Circular map of plasmid from a non-O1/non-O139 *V. cholerae* isolate, highlighting CDS.

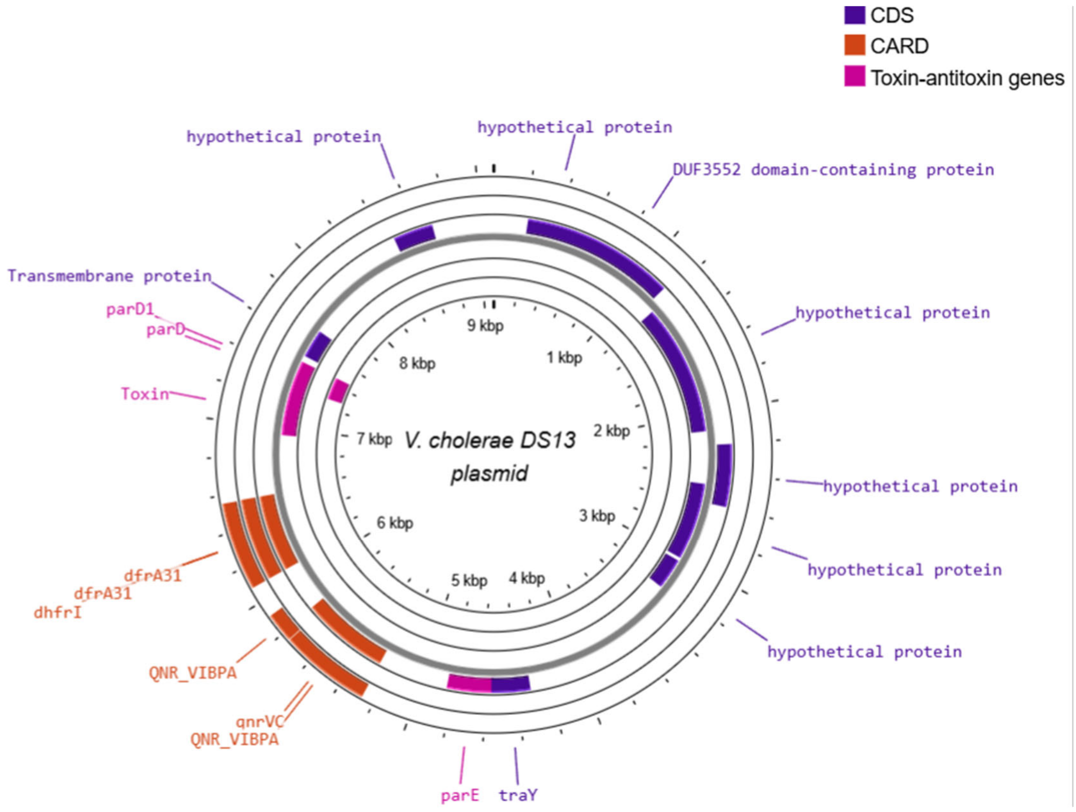

Figure S2: Circular map of a plasmid from a non-O1/non-O139 *V. cholerae* isolate, highlighting antimicrobial resistance determinants, coding DNA sequences and toxin-antitoxin genes

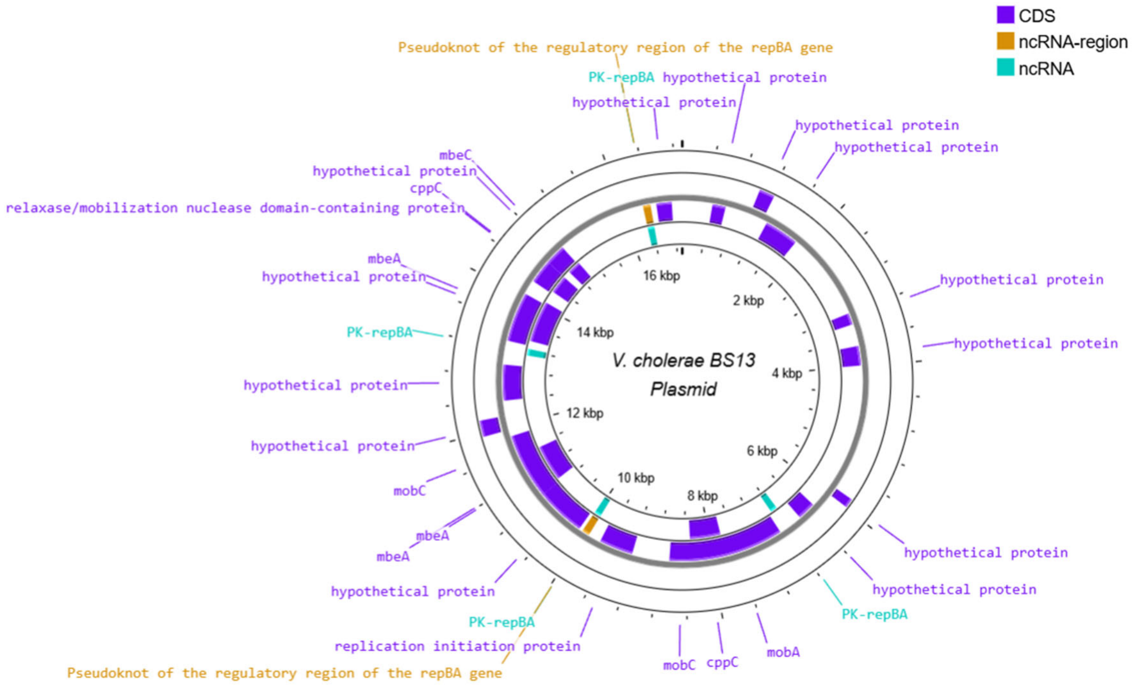

Figure S3: Circular map of an unidentified plasmid from a non-O1/non-O139 *V. cholerae* isolate, highlighting CDS,ncRNA-region and ncRNA

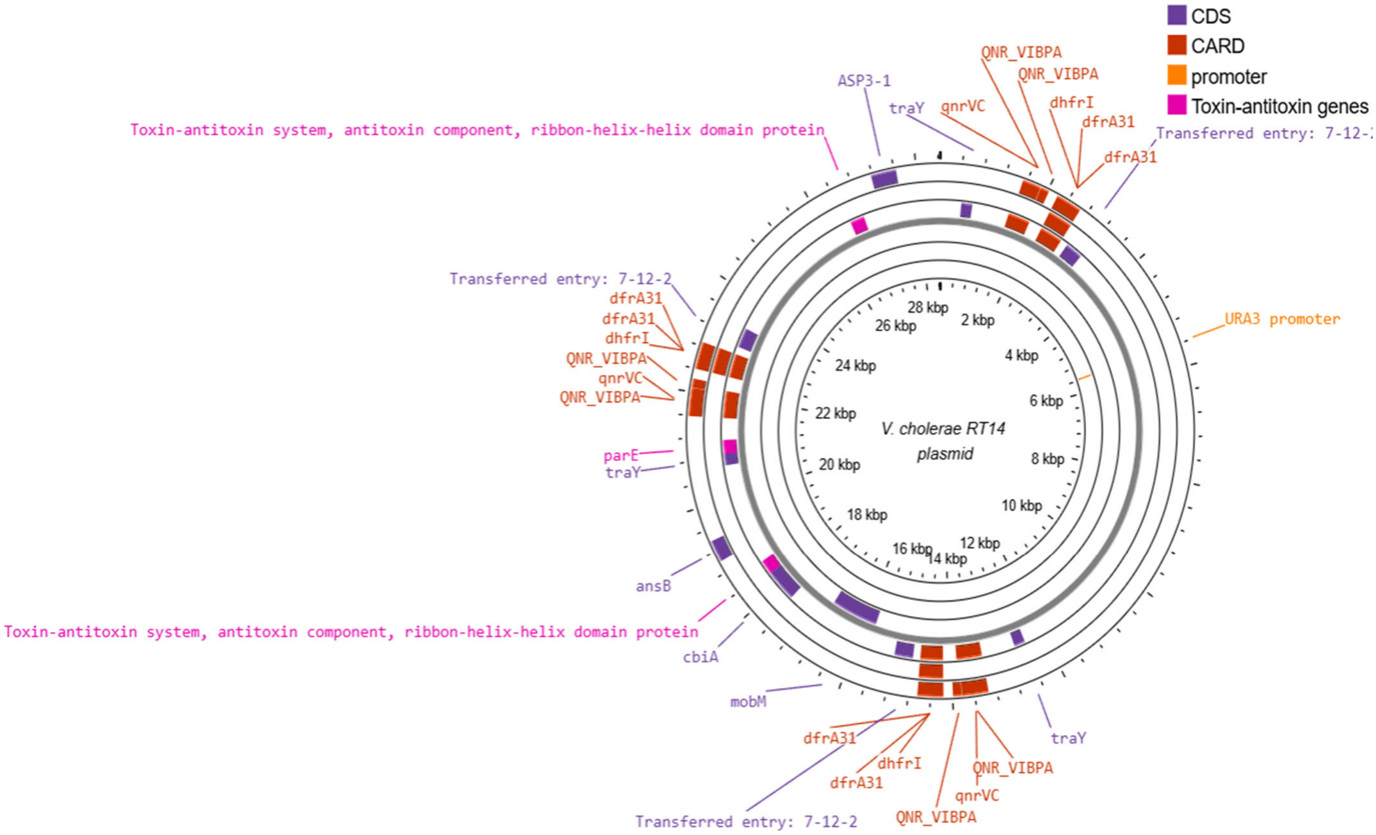

Figure S4: Circular map of a plasmid from a non-O1/non-O139 *V. cholerae* isolate, highlighting CDS, CARD associated with antibiotic resistance and toxin-antitoxin genes

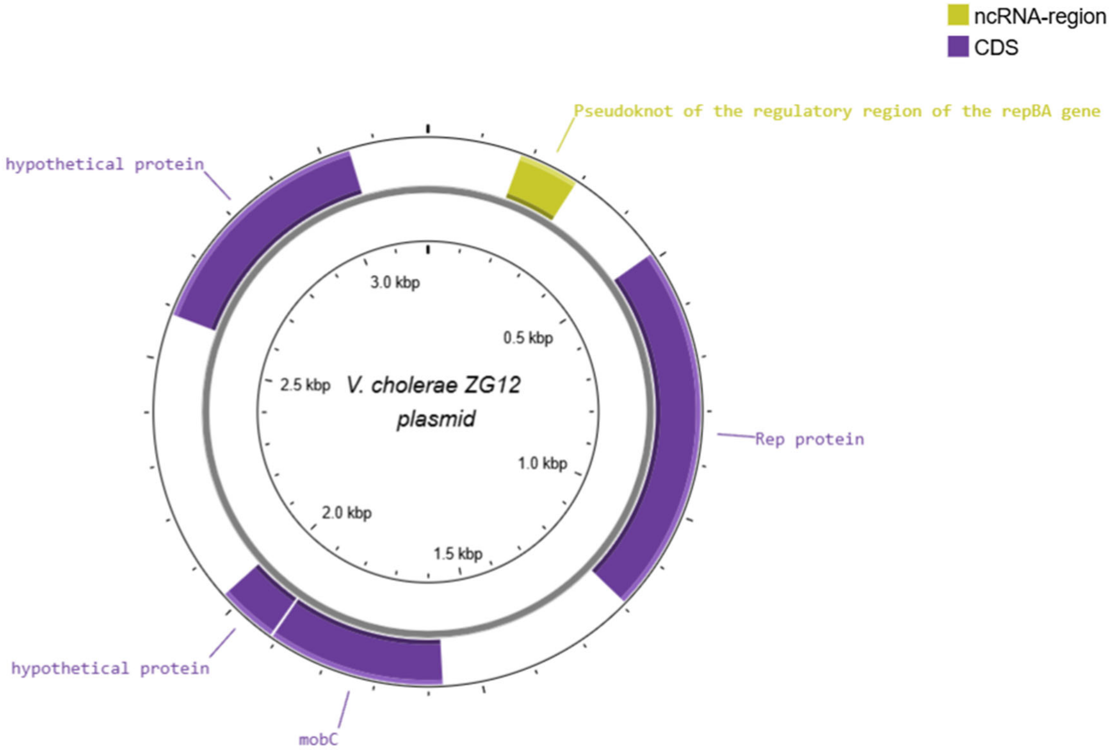

Figure S5: Circular map of an unidentified plasmid from a non-O1/non-O139 *V. cholerae* isolate, highlighting coding DNA sequences and ncRNA-region

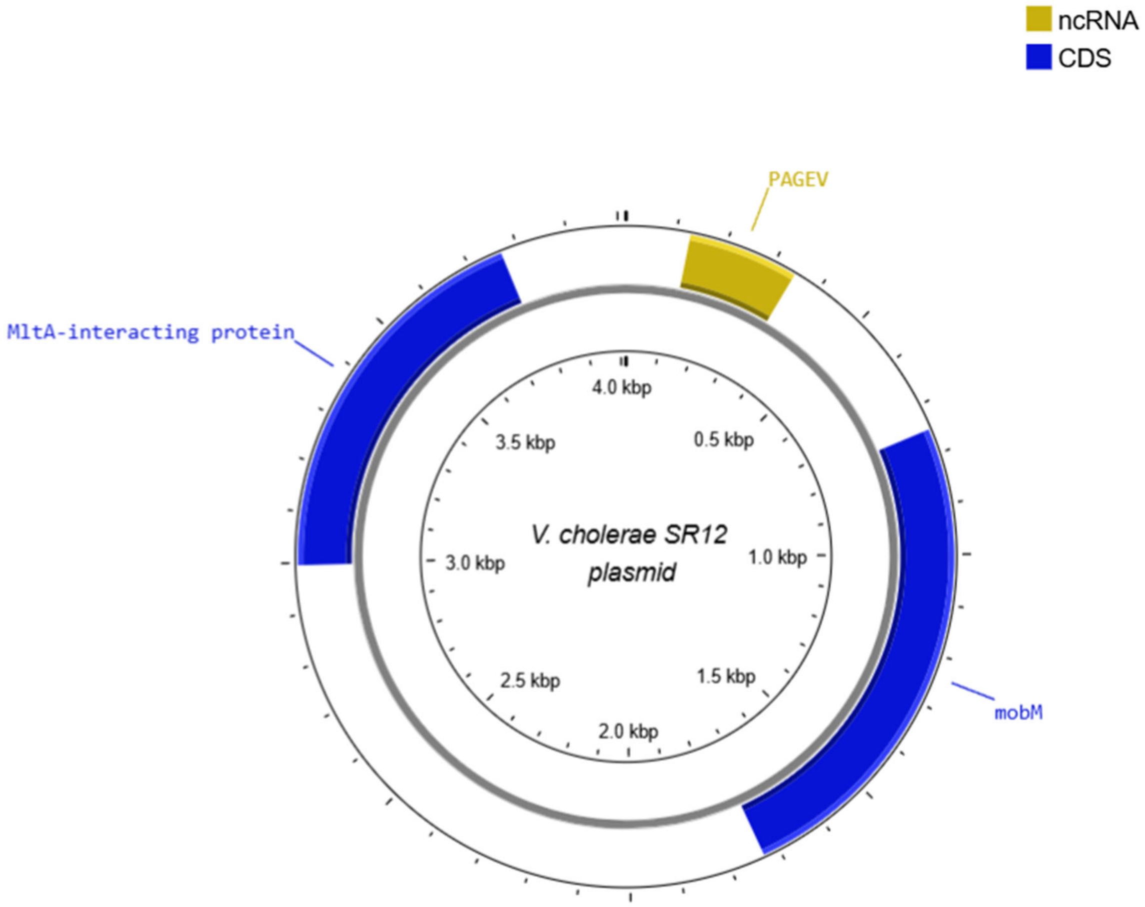

Figure S6: Circular map of a from a non-O1/non-O139 *V. cholerae* isolate, highlighting CDS and ncRNA

Table S3: Comparative analysis of various plasmids detected in NOVC isolates

Isolate ID	Contig	Plasmid sequence with the closest homolog	size (bp)	Percentage identity	putative function
BS12	14	Unnamed plasmid	12,019 bp	99.65%	CDS: Hypothetical proteins
	10	Plasmid PVN84	6,495 bp	99.46%	CDS: *traY*, Hypothetical proteins<br>CARD: Fluoroquinolones (*QNR_VIBPA, qnrVC*) Folate(*dhfrl*)
	9	Plasmid pA	6,480 bp	99.88%	CDS: Hypothetical proteins, *traY*, DUF3552<br>CARD: Fluoroquinolones (*QNR_VIBPA. qnVC*) Folate (*dfrA31, dhfrl*)
BS13	34	Plasmid unnamed	8,230 bp	98.25%	CDS: Hypothetical proteins
	25	Plasmid unnamed	10,050 bp	99.48%	CDS: Hypothetical proteins
	36	Plasmid p2IL	1,191 bp	88.24%	CDS: Hypothetical proteins
	42	Plasmid unnamed	16,906 bp	93.29%	CDS: Hypothetical proteins, pseudoknot of the regulatory region of the *repBA*,

					relaxase/mobilization nuclease domain-containing protein, *mobC,mbeA, mbeC*, replication initiation protein, *mobA, PK-repBA*
	38	Plasmid p2IL	8,062 bp	98.43%	CDS-DNAJ-C domain- containing protein, Hypothetical protein
	37	Plasmid p2IL	1,224 bp	100%	Hypothetical protein
BS14	19	Plasmid unnamed	9.015 bp	97%	*CDS:* hypothetical proteins, *pmCDI, atpF, atpE, atpB, parB,* sporulation initiation inhibitor *protein,soJ,rsmG, mnmG, mnmE,mioC*
	83	Plasmid unnamed	5,003 bp	97.55%	*CDS:* hypothetical proteins, *eco47IIM, haemIIIM, gyrB, parE*
	94	Plasmid pA	8, 241 bp	99.82%	CDS: Hypothetical proteins, L-asparaginase, *mobM, CbiA, traY*<br>*CARD: QNR_VIBPA, QnVC5, dfrA31, dhfrl*<br>Toxin-antitoxin genes: Toxin- antitoxin in component ribbon-helix-helix domain protein
	39	Plasmid unnamed	8,957 bp	99.53%	CDS: Hypothetical proteins, mitA-interacting protein
	12	Plasmid unnamed	11,755 bp	99%	CDS: Hypothetical proteins, *udg, ugd, fabV, Este-VIBMI, lolD, ABC- transporter, ybbA*
RT14	06	Plasmid pA	28, 439 bp	99.83%	CDS: Hypothetical proteins, *traY, CbiA, ASP3-1, ansB, URA3 promoter*<br>*CARD: dhfrl, QNR_VIBPA, dfrA31, qnVC*<br>Toxin-antitoxin genes: antitoxin component ribbon helix-helix domain protein
	05	Plasmid unnamed	3, 903 bp	99.70%	CDS: Hypothetical protein
ZG12	08	Plasmid unnamed	3, 200 bp	93.41%	CDS: Hypothetical protein. Rep protein, pseudoknot for the regulatory region of the repBA gene, mobC
	10	Plasmid p2IL	28, 683 bp	98.48%	CDS: Hypothetical protein
SR12	01	Plasmid unnamed	4,015 bp	99.93%	CDS: mitA- interacting protein, mobM
	03	Plasmid p2IL	12,420 bp	94.20%	CDS: hypothetical protein

Table S4: Summary of antimicrobial resistance profiles of NOVC isolates, showing predicted resistance genes, associated mechanisms, drug classes, and specific antimicrobial agents.

***V. cholerae* Isolates**	**Genes**	**Antibiotics**	**Drug classes**	**Resistance mechanisms**
**BS12**	*qnrVC4*	**CIP**	FQ	Target protection
**BS13**	*BlaCARB-9*	**AMP,** AMO, PIP	PEN	Enzyme degradation

**BS14**	*qnrVC5, dfrA31*	**CIP, TMP**	FQ, FPI	Antibiotic target protection and target replacement
**DS12**	*alph(6)-Id, aph(3")-Ib, floR, qnrVC4, sul2, dfrA6*	**CIP**, STR, CHL, FFC	FQ, AMG, PH, FPI	Efflux pump, enzymatic inactivation, target protection and target replacement
**DS13**	*aph(6)-id, aph(34)-Ib,floR, dfrA31*	**CIP, TMP, SMX,** STR, CHL, TET	AMG, PH, FQ, FPI, TET	Efflux pump, antibiotic inactivation, antibiotic efflux and target protection
**DS14**	*aph(6)-id, aph(3")-Ib, floR, dfrA1*	**SMX, TMP,** STR, CHL, FFC,	AMG, PH, FPI	Antibiotic inactivation, antibiotic efflux, antibiotic replacement
**ZG12**	*BlaCARB-9*	**AMP,** AMO, PIP	PEN	Enzyme degradation
**ZG14**	*blaDHA-9, blaDHA-19, BlaCARB-7, dfrA1*	**AMP,** CTX, CAZ, PIP, AMO	CEP, PEN	Enzymatic degradation and target replacement
**RT12**	*qnrVC4, dfrA31*	**CIP, AMP**, AMO, PIP, TMP	PEN, FQ, FPI	Efflux pump, enzymatic inactivation, target replacement,
**RT14**	*qnrVC5, dfrA31*	**CIP, TMP**	FQ, FPI	Antibiotic target protection and target replacement
**SR12**	*qnrVC4*	**CIP**	FQ	Target protection
**SR14**	*dfrA6, ere(A)*	**TMP**, ERY,	FPI, MAC	Target replacement

Table S5: Distribution of virulence-associated genes in non-O1/non-O139 *Vibrio cholerae* isolates, categorised by functional groups including cytolytic factors, adhesion and colonization factors, secretion systems, biofilm-associated genes, and regulatory/metabolic element.

+	**Virulence factors**												
**Isolates**	**Toxins and cytolytic factors**			**adhesion and colonization**					**secretion systems**				
	hlyA	*rtxA*	*stn*	*mshA*	*ompU*	*tcpA*	*HA/P*	*nagH*	*T3SS*	*T6SS*	*rtxC*	*VPI-1*	*VPI-2*
BS12	+	+	-	+	+	-	-	-	-	-	-	-	-
DS13	+	-	-	+	+	-	-	-	-	-	-	-	-
BS13	+	+	-	+	+	-	-	-	-	-	-	-	-
DS14	+	+	-	+	+	-	-	-	-	-	-	-	-
BS14	+	+	-	-	+	-	-	-	-	-	-	-	-
ZG14	+	+	-	+	+	-	-	-	-	-	-	-	-
RT14	+	-	-	+	+	-	-	-	-	-	-	-	-
SR14	+	-	-	-	-	-	-	-	-	-	-	-	-
DS12	+	-	-	+	+	-	-	-	-	-	-	-	-
ZG12	+	-	-	+	+	-	-	-	-	-	-	-	-
RT12	+	-	-	+	+	-	-	-	-	-	-	-	-
SR12	+	-	-	+	+	-	-	-	-	-	-	-	-
	**Virulence factors**												
**Isolates**	**Biofilm**			**Regulatory and metabolic**									

	VSP1/VSP 2	*als*	*makA*	*toxR*	*VSPR*	*rfbv*	*rfbc*	*trkH*	*tna A*	*gyrA*	*gyrB*
BS12	-	-	-	-	-	-	-	+	+	+	+
DS13	-	-	-	-	-	-	-	+	+	+	+
BS13	-	-	-	-	-	-	-	+	+	+	+
DS14	-	-	-	-	-	-	-	+	+	+	+
BS14	-	-	-	-	-	-	+	+	+	+	+
ZG14	-	-	+	-	+	-	-	+	+	-	+
RT14	-	-	-	-	-	-	-	+	+	+	+
SR14	-	-	-	-	-	-	-	+	+	+	+
DS12	-	-	-	-	-	-	-	+	+	+	+
ZG12	-	-	-	-	-	-	-	+	+	+	+
RT12	-	-	-	-	-	-	+	+	+	+	+
SR12	-	-	-	-	-	-	-	+	+	+	+

Supplementary S3: Whole genome maps

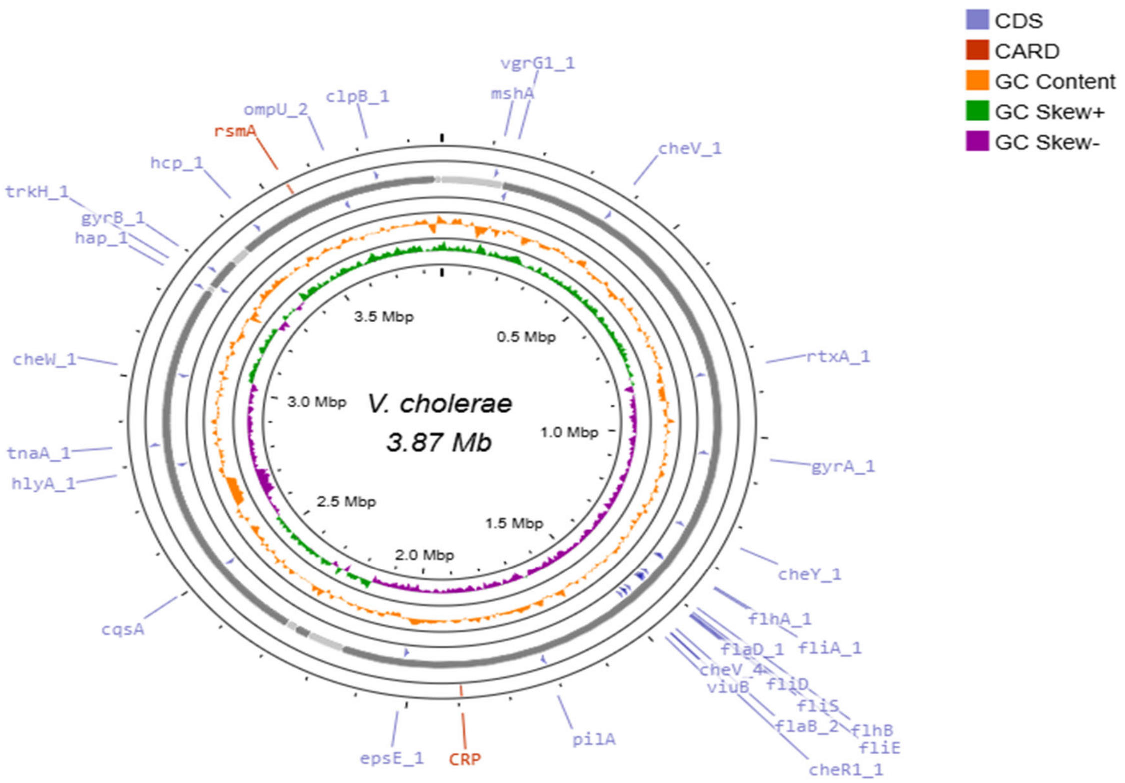

Figure S7: Complete genome annotation of the non-O1/non-O139 *V. cholerae* isolate BS12. The innermost rings represent the G+C content and GC skew, while the outermost rings display coding DNA sequences (CDS) and antimicrobial resistance genes identified using the Comprehensive Antibiotic Resistance Database (CARD). CDS homologous to known virulence genes (*hlyA*, *gyrA/B*, outer membrane proteins (*ompW*, *ompV*, *ompA*, *ompR* and *ompH*), *trkH* and *tnaA*) are highlighted,

alongside CARD-annotated resistance genes (*rsmA, CRP*). This genomic map provides a visual overview of both the virulence potential and antimicrobial resistance profile of BS12.

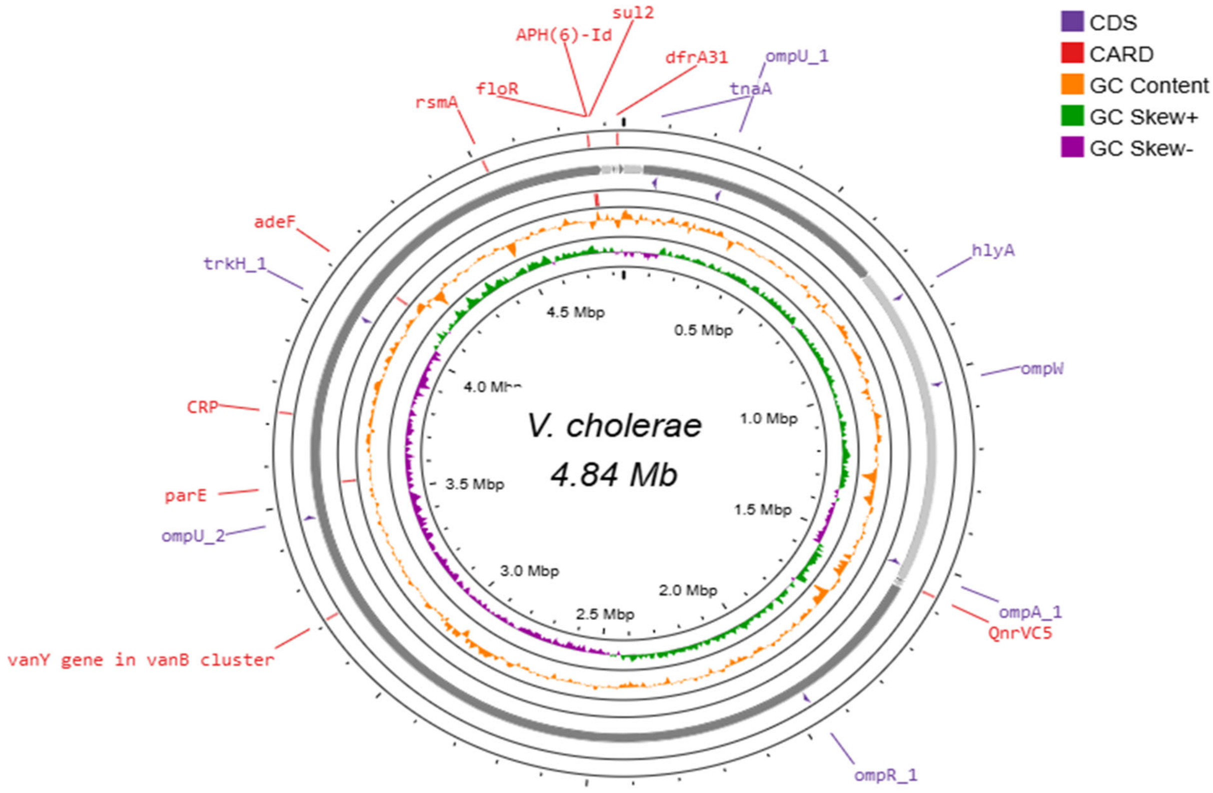

Figure S8: Complete genome annotation of the non-O1/non-O139 *V. cholerae* isolate DS13. The innermost rings represent the G+C content and GC skew, while the outermost rings display coding DNA sequences (CDS) and antimicrobial resistance genes identified using the Comprehensive Antibiotic Resistance Database (CARD). CDS homologous to known virulence genes (*hlyA, gyrA/B* and outer membrane proteins (*ompW, ompR, ompA* and *ompU), trkH* and *tnaA)* are highlighted, alongside CARD-annotated resistance genes (*rsmA, vanY, CRP, adeF, dfrA31, sul2, APH (3")-Ib, APH (6)-Id, floR, CRP and QnVC5*). This genomic map provides a visual overview of both the virulence potential and antimicrobial resistance profile of DS13.

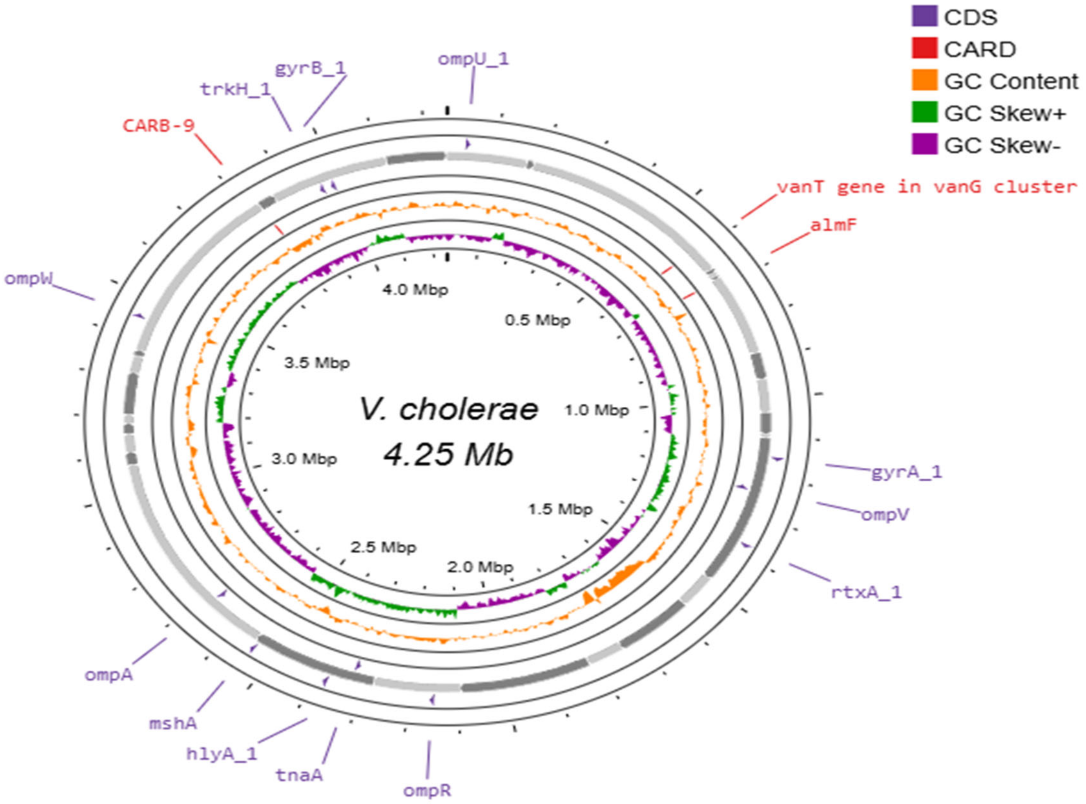

Figure S9: Complete genome annotation of the non-O1/non-O139 *V.cholerae* isolate BS13. The innermost rings represent the G+C content and GC skew, while the outermost rings display coding DNA sequences (CDS) and antimicrobial resistance genes identified using the Comprehensive Antibiotic Resistance Database (CARD). CDS homologous to known virulence genes **(***hlyA, gyrA/B, mshA, rtxA, outer membrane proteins (ompW, ompU, ompA, ompV, ompR), trkH, tnaA))* are highlighted, alongside CARD-annotated resistance genes (*vanT, almF, CARB-9*). This genomic map provides a visual overview of both the virulence potential and antimicrobial resistance profile of BS13.

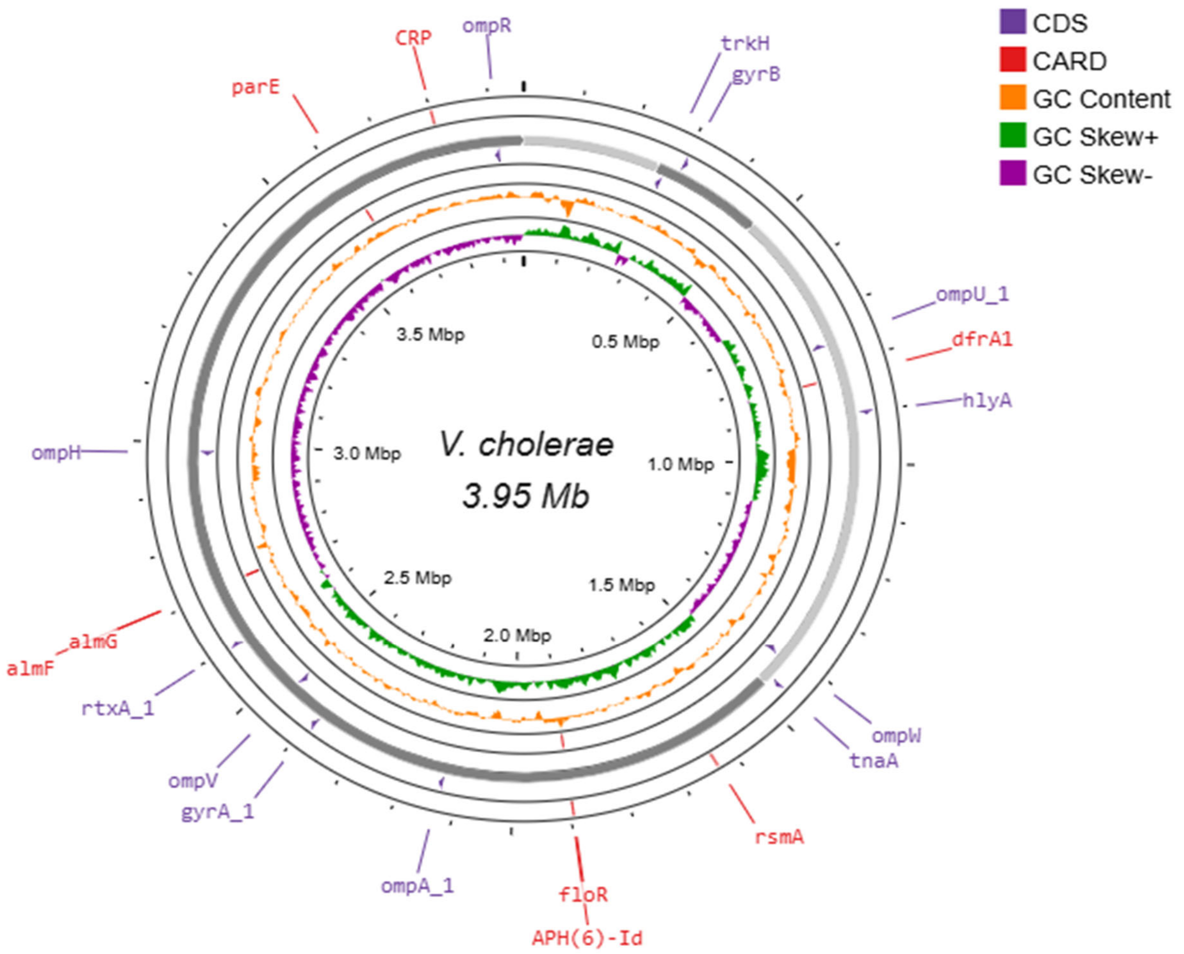

Figure S10: Complete genome annotation of the non-O1/non-O139 *V. cholerae* isolate DS14. The innermost rings represent the G+C content and GC skew, while the outermost rings display coding DNA sequences (CDS) and antimicrobial resistance genes identified using the Comprehensive Antibiotic Resistance Database (CARD). CDS homologous to known virulence genes (*(hlyA, gyrA/B, rtxA and outer membrane proteins (ompW, ompH, ompV, ompA, ompU and ompR), trkH, tnaA)* are highlighted, alongside CARD-annotated resistance genes (*floR, CRP, dfrA1, rsmA, almF, almG, APH (6)-Id and parE*). This genomic map provides a visual overview of both the virulence potential and antimicrobial resistance profiles of DS14.

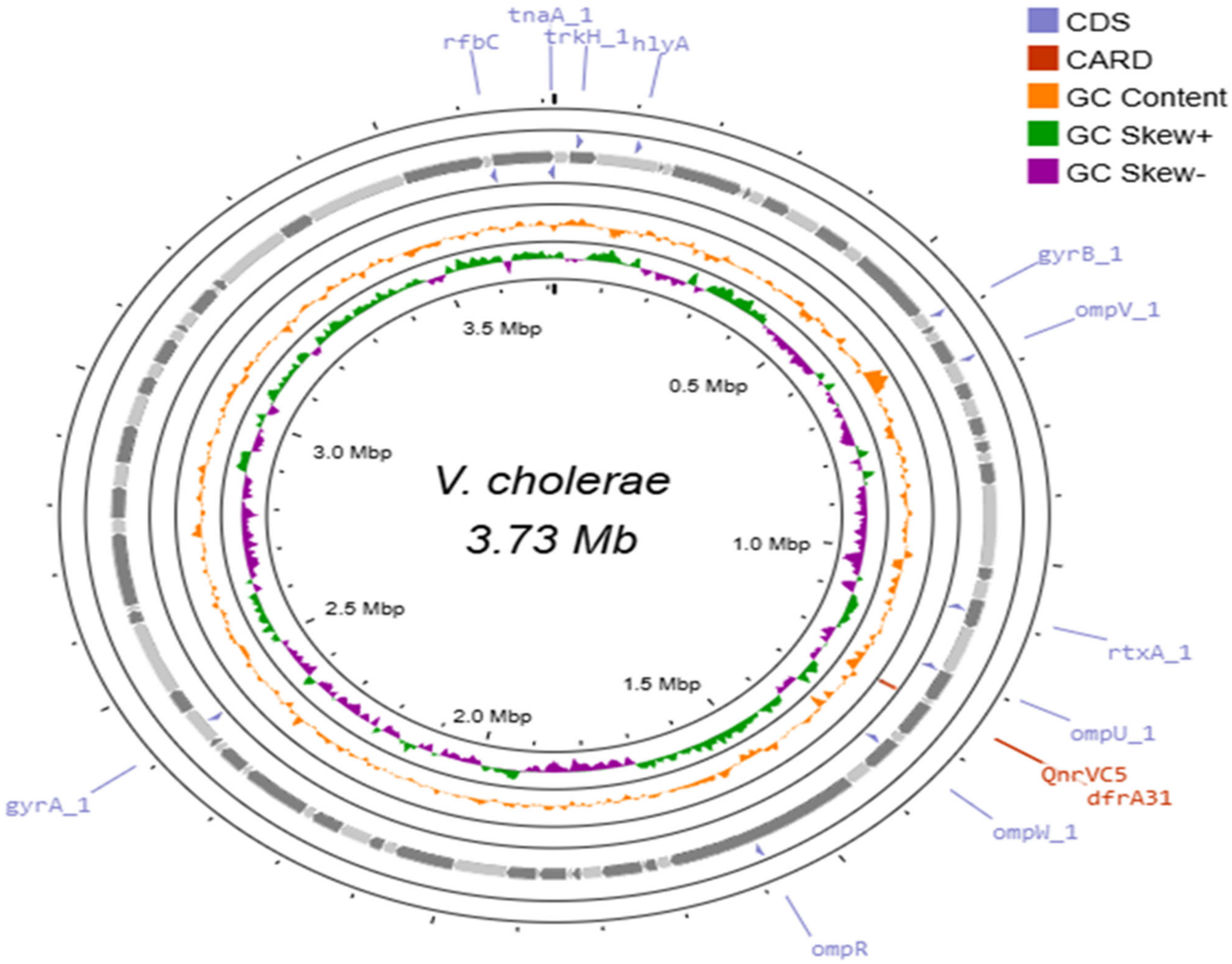

Figure S11: Complete genome annotation of the non-O1/non-O139 *V. cholerae* isolate BS14. The innermost rings represent the G+C content and GC skew, while the outermost rings display coding DNA sequences (CDS) and antimicrobial resistant genes identified using the Comprehensive Antibiotic Resistance Database (CARD). CDS homologous to known virulence genes (*hlyA*, *gyrA/B*, *rtxA*, *rfbC*, outer membrane proteins (*ompW*, *ompR*, *ompU*, *ompV*,), *trkH*, *tnaA*, *tdh)* are highlighted, alongside CARD-annotated resistance genes (*QnrVC5*, *dfA31*). This genomic map provides a visual overview of both the virulence potential and antimicrobial resistance profile of BS14.

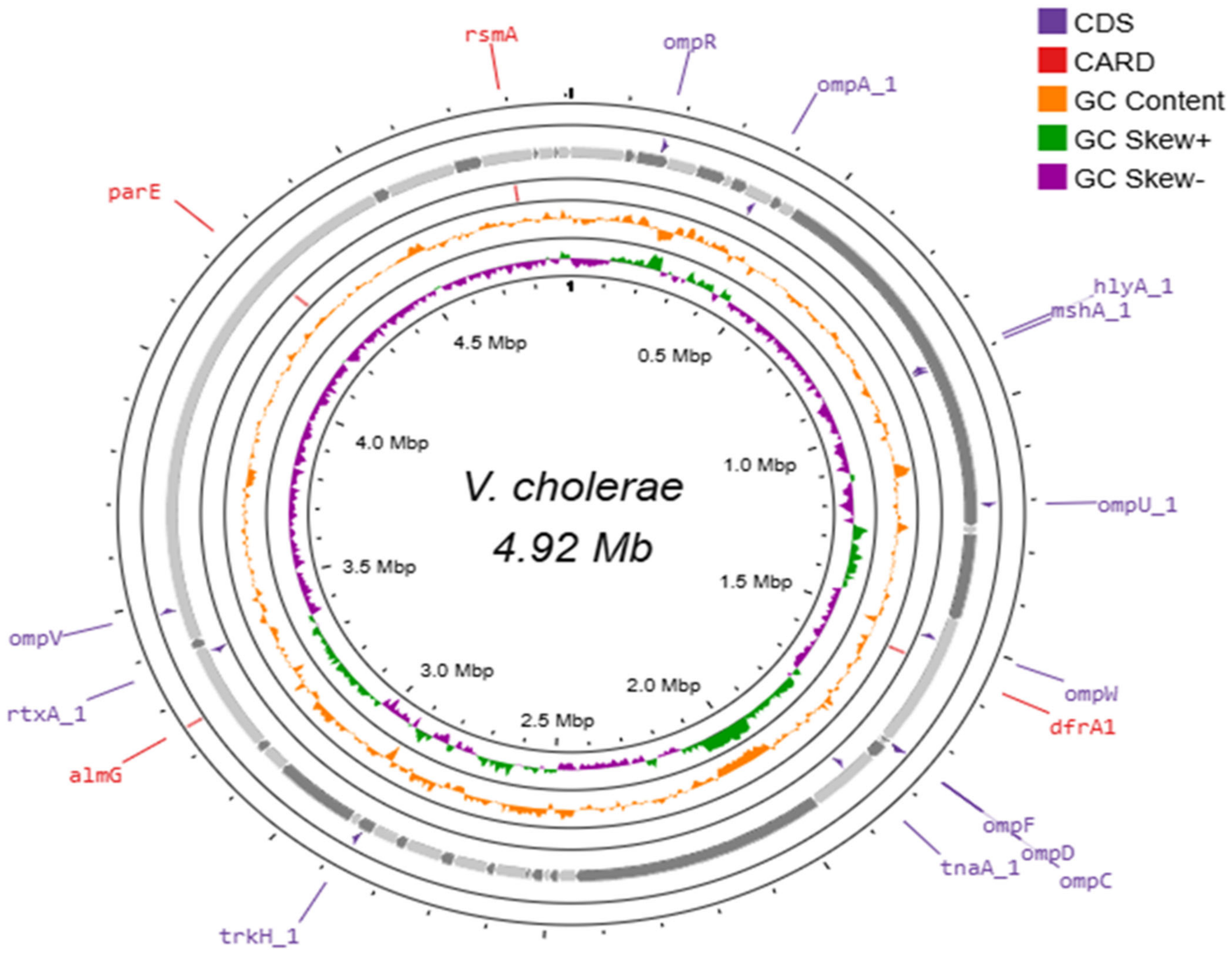

Figure S12: Complete genome annotation of the non-O1/non-O139 *V. cholerae* isolate ZG14. The innermost rings represent the G+C content and GC skew, while the outermost rings display coding DNA sequences (CDS) and antimicrobial resistance genes identified using the Comprehensive Antibiotic Resistance Database (CARD). CDS homologous to known virulence genes (*hlyA, gyrA/B, rtxA, mshA* and outer membrane proteins (*ompW, ompR, ompV, ompU, ompA, ompF, ompD* and *ompC), trkH, tnaA)* are highlighted, alongside CARD-annotated resistance genes (*rsmA, almG, dfrA1, parE*). This genomic map provides a visual overview of both the virulence potential and antimicrobial resistance profile of ZG14.

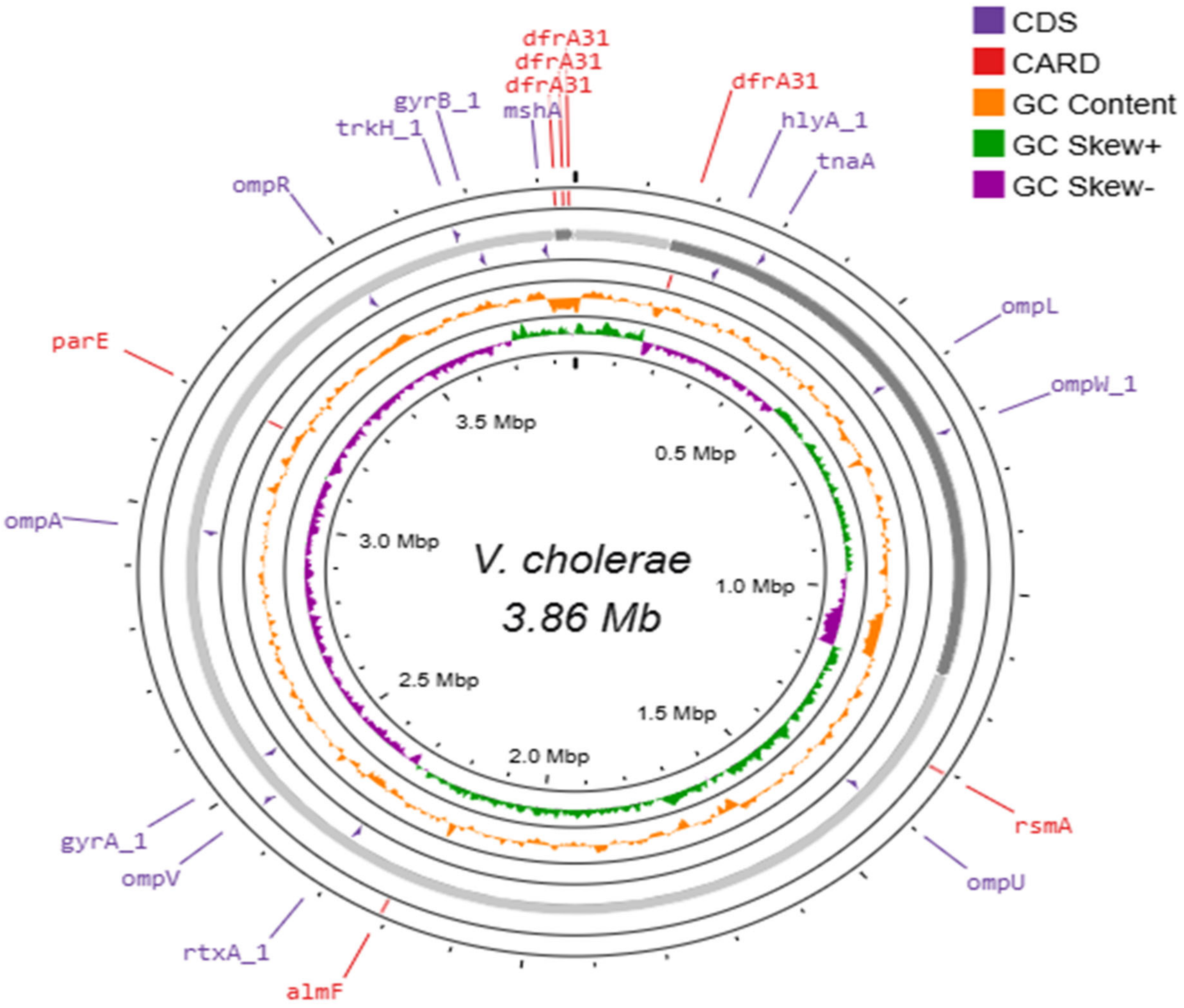

Figure S13: Complete genome annotation of the non-O1/non-O139 *V.cholerae* isolate RT14. The innermost rings represent the G+C content and GC skew, while the outermost rings display coding DNA sequences (CDS) and antimicrobial resistance genes identified using the Comprehensive Antibiotic Resistance Database (CARD). CDS homologous to known virulence genes (*hlyA, gyrA/B, mshA*, *rtxA*, outer membrane proteins (*ompW, ompR, ompV, ompU, ompA and ompL), trkH, tnaA, tdh)* are highlighted, alongside CARD-annotated resistance genes (*rsmA, almF, dfrA31*). This genomic map provides a visual overview of both the virulence potential and antimicrobial resistance profile of RT14

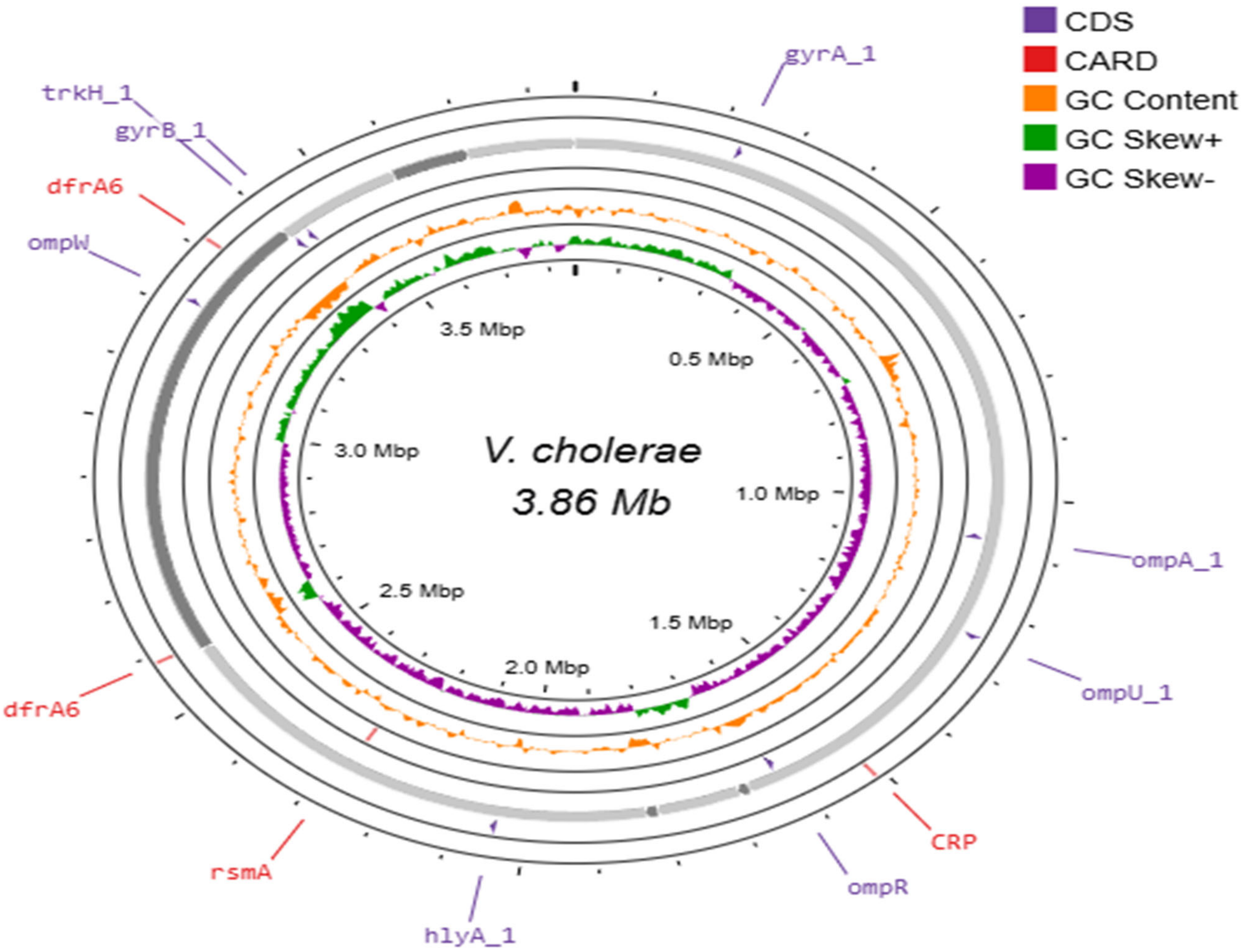

Figure S14: Complete genome annotation of the non-O1/non-O139 *V. cholerae* isolate SR14. The innermost rings represent the G+C content and GC skew, while the outermost rings display coding DNA sequences (CDS) and antimicrobial resistance genes identified using the Comprehensive Antibiotic Resistance Database (CARD). CDS homologous to known virulence genes genes (*hlyA, gyrA/B,* outer membrane proteins (*ompA, ompW, ompR, ompP and ompU)* and *trkH))* are highlighted, alongside CARD-annotated resistance genes (*rsmA, CRP, dfrA6*). This genomic map provides a visual overview of both the virulence potential and antimicrobial resistance profile of SR14.

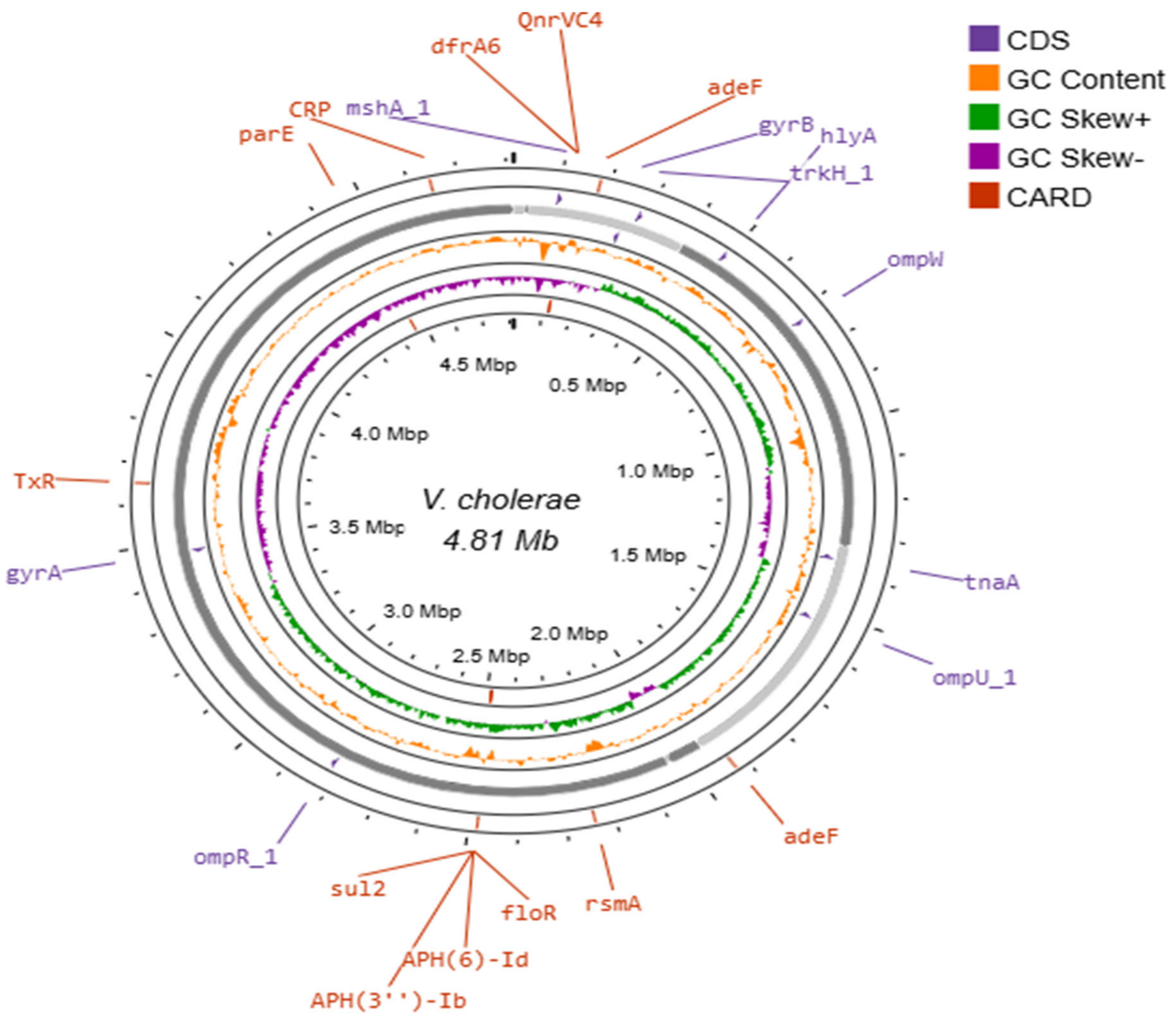

Figure S15: Complete genome annotation of the non-O1/non-O139 *V. cholerae* isolate DS12. The innermost rings represent the G+C content and GC skew, while the outermost rings display coding DNA sequences (CDS) and antimicrobial resistance genes identified using the Comprehensive Antibiotic Resistance Database (CARD). CDS homologous to known virulence genes genes (*hlyA, gyrA/B, mshA, outer membrane proteins (ompW, ompA, ompV, ompU, and ompA), trkH, tnaA)* are highlighted, alongside CARD-annotated resistance genes (*rsmA, parE CRP, QnrVC4, adeF, rsmA, floR, APH (6)-Id, sul2, TXR*). This genomic map provides a visual overview of both the virulence potential and antimicrobial resistance profile of DS12.

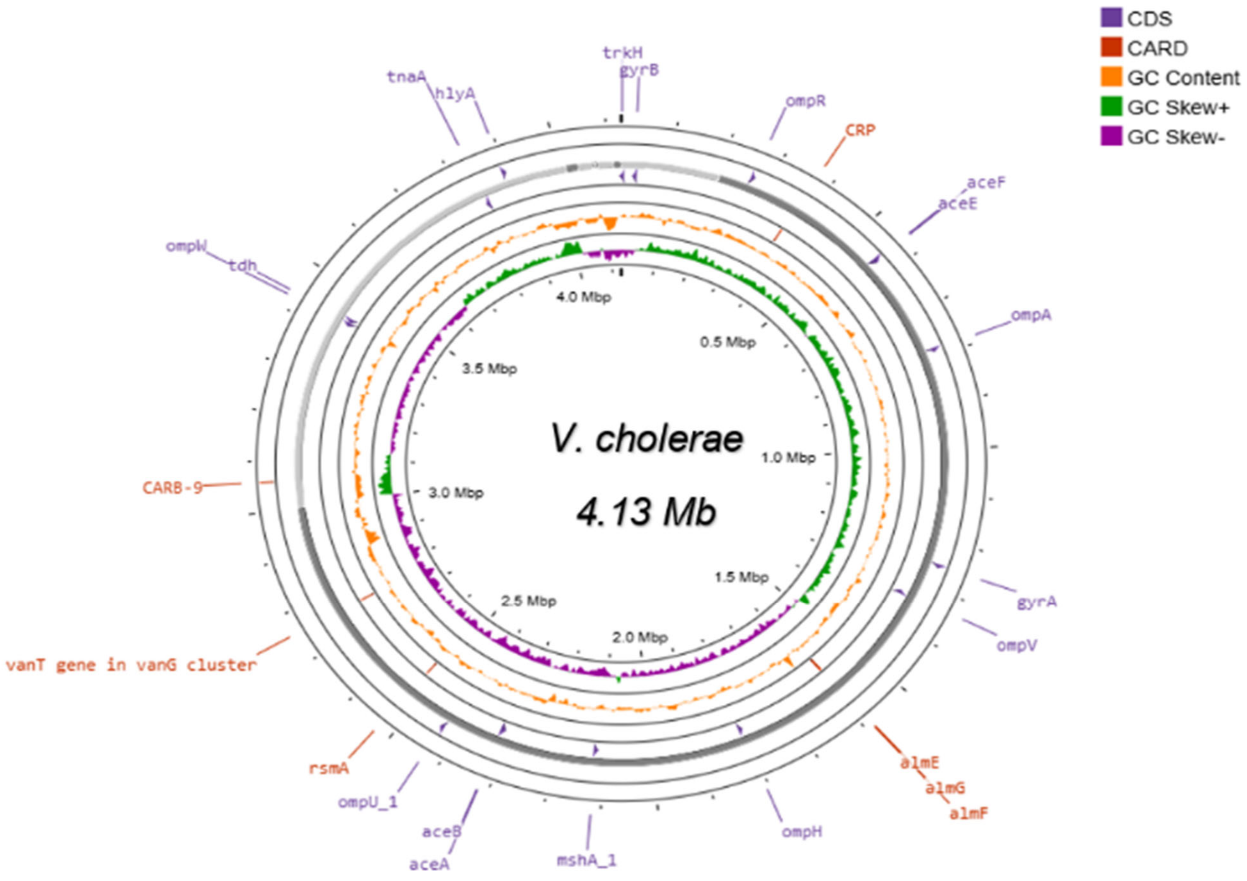

Figure S16: Complete genome annotation of the non-O1/non-O139 *V. cholerae* isolate ZG12. The innermost rings represent the G+C content and GC skew, while the outermost rings display coding DNA sequences (CDS) and antimicrobial resistance genes identified using the Comprehensive Antibiotic Resistance Database (CARD). CDS homologous to known virulence genes (*hlyA, ace, mshA, gyrA/B and outer membrane proteins (ompA, ompR, ompU, ompW, ompH and ompV), trkH, tnaA and tdh)* are highlighted, alongside CARD-annotated resistance genes (*rsmA, vanT, CRP, almE, almG and almF*). This genomic map provides a visual overview of both the virulence potential and antimicrobial resistance profile of ZG12.

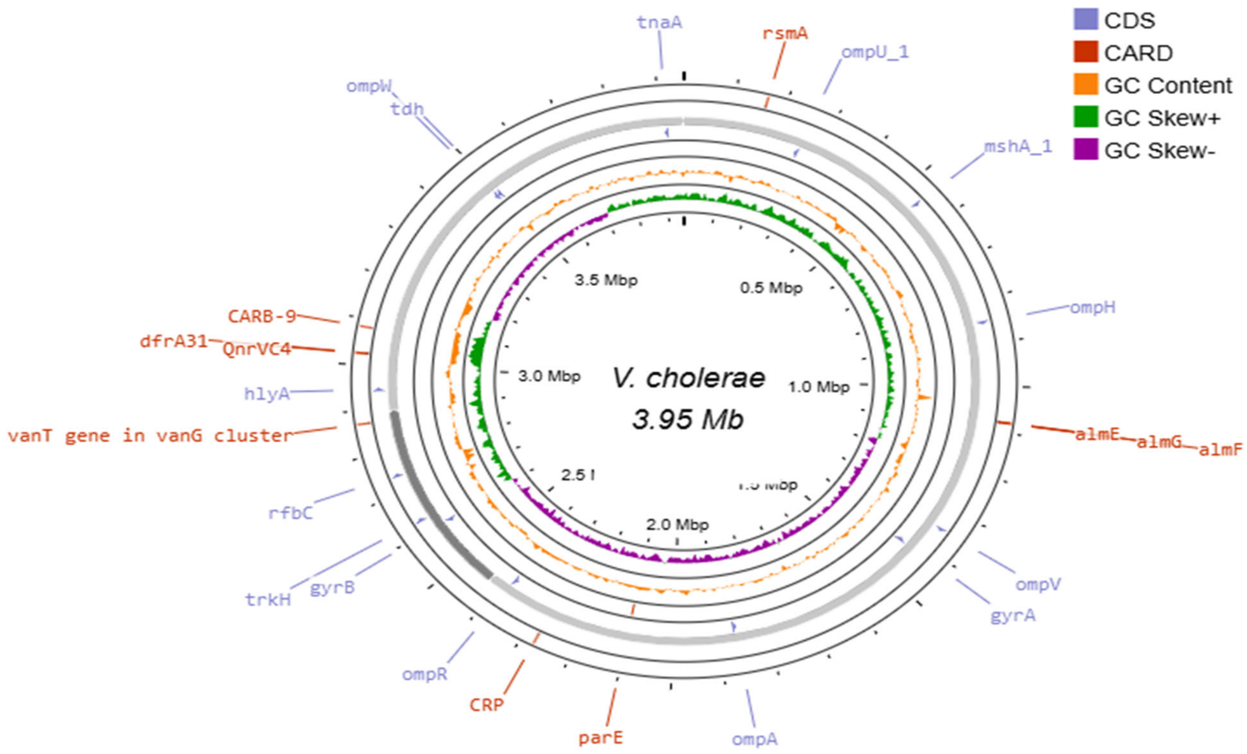

Figure S17: Complete genome annotation of the non-O1/non-O139 *V. cholerae* isolate RT12. The innermost rings represent the G+C content and GC skew, while the outermost rings display coding DNA sequences (CDS) and antimicrobial resistance genes identified using the Comprehensive Antibiotic Resistance Database (CARD). CDS homologous to known virulence genes (*hlyA, ace, gyrA/B, mshA, rfbC, outer membrane proteins (ompW, ompA, ompV, ompU, ompH and ompR), trkH, tnaA, tdh)* are highlighted, alongside CARD-annotated resistance genes (*vanT, CARB-9, dfrA31, QnrVC4, almF, almE, almG*). This genomic map provides a visual overview of both the virulence potential and antimicrobial resistance profile of RT12.

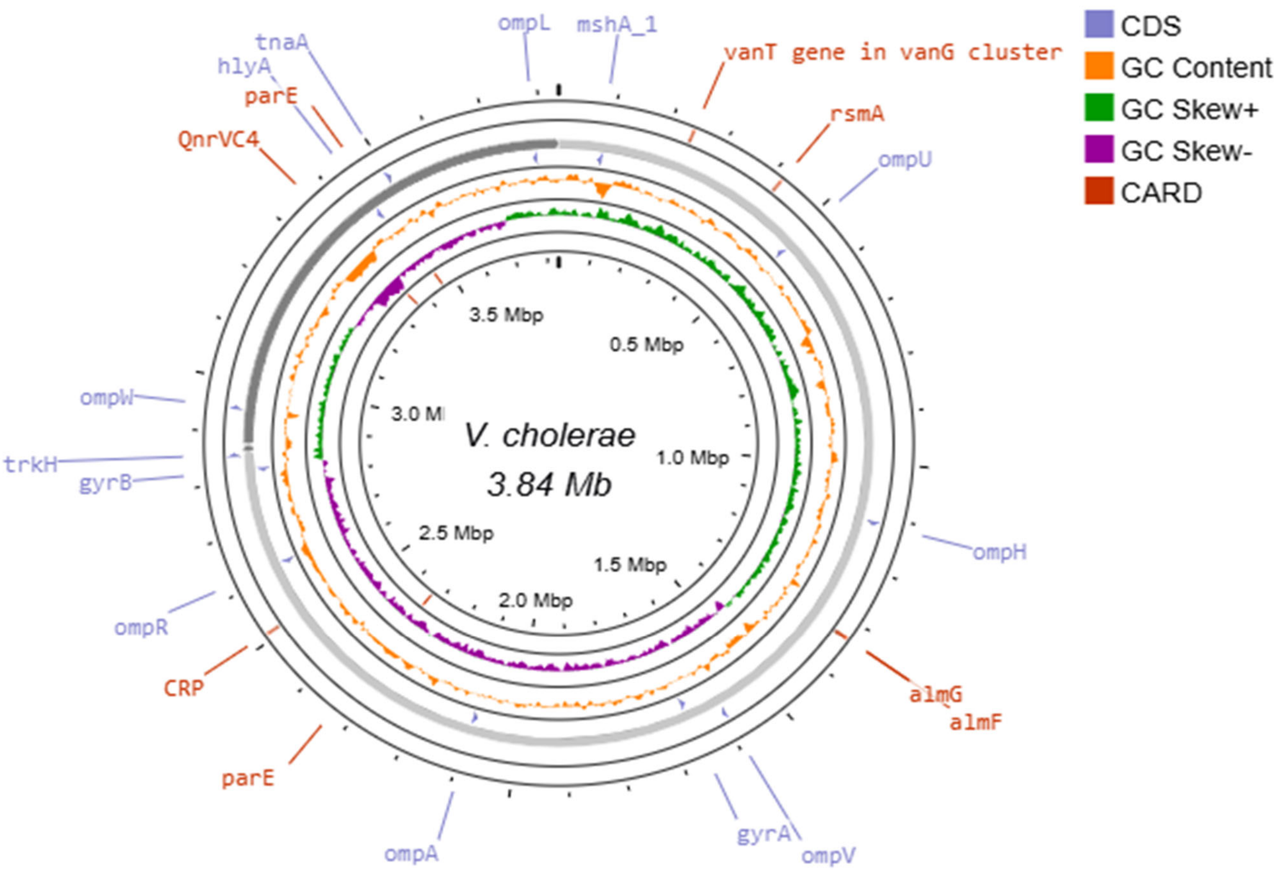

Figure S18: Complete genome annotation of the non-O1/non-O139 *V. cholerae* isolate SR12. The innermost rings represent the G+C content and GC skew, while the outermost rings display coding DNA sequences (CDS) and antimicrobial resistance genes identified using the Comprehensive Antibiotic Resistance Database (CARD). CDS homologous to known virulence genes *(hlyA, ace, gyrA/B, mshA, outer membrane proteins (ompA, ompR, ompU, ompL, ompH, ompV and ompW), trkH, tnaA, tdh)* are highlighted, alongside CARD-annotated resistance genes (*rsmA, vanT, QnrVC4, CRP, almF, almG*). This genomic map provides a visual overview of both the virulence potential and antimicrobial resistance profile of SR12.
